# Prospective Field Validation of the START:AV in a Dutch Secure Youth Care Sample

**DOI:** 10.1177/10731911211063228

**Published:** 2021-12-15

**Authors:** Tamara L. F. De Beuf, Vivienne de Vogel, Nick J. Broers, Corine de Ruiter

**Affiliations:** 1 Ottho Gerhard Heldring Institution, Zetten, The Netherlands; 2 Maastricht University, The Netherlands; 3 De Forensische Zorgspecialisten, Utrecht, The Netherlands

**Keywords:** START:AV, risk assessment, field validity, predictive validity, adolescent, strengths, adverse outcomes

## Abstract

The Short-Term Assessment of Risk and Treatability: Adolescent Version (START:AV) is a risk assessment instrument for adolescents that estimates the risk of multiple adverse outcomes. Prior research into its predictive validity is limited to a handful of studies conducted with the START:AV pilot version and often by the instrument’s developers. The present study examines the START:AV’s field validity in a secure youth care sample in the Netherlands. Using a prospective design, we investigated whether the total scores, lifetime history, and the final risk judgments of 106 START:AVs predicted inpatient incidents during a 4-month follow-up. Final risk judgments and lifetime history predicted multiple adverse outcomes, including physical aggression, institutional violations, substance use, self-injury, and victimization. The predictive validity of the total scores was significant only for physical aggression and institutional violations. Hence, the short-term predictive validity of the START:AV for inpatient incidents in a residential youth care setting was partially demonstrated and the START:AV final risk judgments can be used to guide treatment planning and decision-making regarding furlough or discharge in this setting.

In the past three decades, much effort has been invested in the development of structured, empirically based risk assessment instruments (Heilbrun et al., 2021). They have been found to produce moderate levels of predictive accuracy (Fazel et al., 2012; Yang et al., 2010) and have been adopted widely in forensic, correctional, and court settings across the globe (Singh et al., 2014). Risk assessment instruments are designed to guide risk management decision-making, such as determining the appropriate level of supervision and selecting interventions that address a person’s criminogenic needs (Bonta & Andrews, 2017). Structured risk assessment has become indispensable in criminal justice and forensic mental health settings, both for adult and adolescent populations (see Douglas & Otto, 2021, for an up-to-date review of the state of the field).

## Developmentally Appropriate Risk Assessment

Especially for young people, timely and effective risk assessment is important from a rehabilitative perspective, to minimize the risk of persistent antisocial or otherwise problematic life trajectories (Lodewijks et al., 2010). To facilitate this goal, instruments need to be attuned to adolescent development and require consideration of specific features (J. L. Viljoen et al., 2012). First, adolescent risk assessment instruments should include developmentally appropriate factors. Although many risk factors for adults are also relevant for adolescents, they may manifest differently or their relevance may vary depending on the developmental stage (Borum et al., 2021). For example, although employment may be relevant to adolescents who hold side jobs or internships, the emphasis will be more on school functioning compared to adults (Bonta & Andrews, 2017). Similarly, resources of the family (vs. the adolescent’s personal resources) will be more relevant to adolescents because most of them are still living with caregivers rather than independently. Second, in addition to developmentally informed risk factors, protective factors or strengths are particularly important for risk assessment and risk management in adolescents (Lodewijks et al., 2010). Strengths, such as positive relationships with prosocial adults or motivation for school, are empirically linked with criminal desistance in juvenile offenders (Lodewijks et al., 2010; Shepherd et al., 2018). Finally, other developmentally relevant features for adolescent risk assessment are the inclusion of dynamic, changeable factors and regular re-assessment, also over the short term. Adolescence is a period of rapid developmental changes: neurologically, physically, and mentally. Consequently, the “shelf life” of adolescent risk assessments will be shorter than risk assessments for adults, making reassessments with short time intervals more appropriate (Vincent & Grisso, 2005). Furthermore, for short-term predictions, dynamic factors, such as antisocial attitudes, have been found to be more accurate predictors than static, historical factors, such as age at first offense (Chu et al., 2011). Examples of dynamic factors that are empirically associated with juvenile reoffending are delinquent peers, substance abuse, hostile beliefs, and poor school adjustment or academic achievement (McGrath & Thompson, 2012). In sum, developmentally appropriate dynamic risk factors and strengths are essential to adolescent risk assessment.

There are several adolescent risk assessment instruments that take these features into account, to varying extents (see Hoge & Andrews, 2010). The present paper focuses on one of these instruments: the Short-Term Assessment of Risk and Treatability: Adolescent Version (START:AV; J. L. Viljoen et al., 2014). Specifically, we will examine its predictive performance in a field study, using a residential youth care sample. Before we describe the present study, we briefly introduce the instrument and the available research on its predictive validity.

## The Short-Term Assessment of Risk and Treatability: Adolescent Version

The START:AV is derived from a validated risk assessment instrument for adults in (forensic) mental health and justice settings: the Short-Term Assessment of Risk and Treatability (START; Webster et al., 2009; for a review see O’Shea & Dickens, 2014). The adolescent version is developed for use with boys and girls between the ages of 12 and 18 in a range of settings, including juvenile justice and (forensic) mental health settings. As the instrument’s name suggests, risk is (re)assessed in the short term, preferably every 3 months. This rapid reassessment cycle is compatible with the instrument’s focus on dynamic factors. Each factor is rated twice: as a protective factor (strength) and as a risk factor (vulnerability). Having an equal number of risk and protective factors is a distinguishing characteristic of the START:AV; most other adolescent risk assessment instruments primarily focus on risk factors (J. L. Viljoen et al., 2012). The strengths and vulnerabilities included in the START:AV are developmentally informed: the item descriptors are appropriate for adolescence (e.g., relationships with caregivers instead of intimate partner relationships) and they reflect the social ecosystems that are important to juveniles (e.g., school, family, peers, community).

Arguably, the START:AV’s most notable feature is the inclusion of multiple risk domains or adverse outcomes. That is, in addition to the risk of violence and criminal offending, the instrument evaluates the risk of substance abuse, unauthorized absences, suicide, self-injury, victimization, and health neglect. Research has shown that adverse outcomes tend to co-occur because they partly share the same predictors (Farrell et al., 2000) or because having one adverse outcome increases the risk of another. For example, impulsivity is a risk factor for multiple adverse outcomes, including substance abuse (Felton et al., 2020) and, in turn, substance abuse is associated with an increased risk of violence and suicide (Becker & Grilo, 2007). Thus, instead of relying on multiple measures for various adverse outcomes, risk factors and adverse outcomes are combined in one instrument (Webster et al., 2006).

## START:AV Predictive Validity Studies

To our knowledge, there have been six studies on the predictive validity of the START:AV thus far, as described in publications (Sher et al., 2017; J. L. Viljoen et al., 2012) and presentations (Johnson et al., 2014; J. L. Viljoen, Gray, et al., 2015; J. L. Viljoen, Shaffer, et al., 2015; S. Viljoen, 2014). The studies were conducted in a correctional facility (Johnson et al., 2014), medium security adolescent psychiatric facility (Sher et al., 2017), American Indian/Alaska Native (AI/AN) residential treatment center (S. Viljoen, 2014), and in the context of community probation supervision (J. L. Viljoen, Beneteau, et al., 2012; J. L. Viljoen, Gray, et al., 2015; J. L. Viljoen, Shaffer, et al., 2015), with sample sizes ranging from 30 to 90 adolescents. All studies except the study by Johnson et al. involved a 3-month follow-up period for the adverse outcomes. The average follow-up period for Johnson et al. (2014) was 4.7 months (*SD* = 3.1), ranging between 8 days and 12 months. J. L. Viljoen, Gray, et al. (2015) additionally measured violence over short-term (3–6 months), medium-term (6–12 months, 12–24 months), and long-term (24–60 months) follow-up periods, whereas J. L. Viljoen, Shaffer, et al. (2015) additionally assessed violence and any offending after 12 months.

Two of these studies (Johnson et al., 2014; Sher et al., 2017) are field studies in which the START:AV assessments were conducted by practitioners in the context of clinical decision-making. Four studies were (co)authored by the instrument’s developers and all studies relied on the START:AV pilot version, a concise, 10-page rating guide containing the item anchors (Nicholls et al., 2010). Note that the final version of the START:AV (J. L. Viljoen et al., 2014) includes three additional items and, among other changes, the item anchors were reformulated (see Bhanwer et al., 2016).

For a comprehensive review of these validity studies, we refer to the START:AV annotated bibliography (Bhanwer et al., 2016). To our knowledge, there are no other studies on the predictive validity of the START:AV besides those discussed in the annotated bibliography. In sum, for a 3-month follow-up period, evidence was found for the predictive validity of both vulnerability and strength total scores for multiple adverse outcomes, including violence (physical and verbal), nonviolent offending, substance use (street drugs), unauthorized absences, and bullying. In addition, the vulnerability total score was predictive of victimization and health neglect. The final risk judgments were predictive of violent offending (physical and verbal), nonviolent offending (property damage), substance use (alcohol, marihuana, and street drugs), self-injury, and bullying.

## Present Study

In the present study, we aim to extend the empirical literature by validating the START:AV for a new target group: youth in secure residential care. Given that the START:AV assessments are conducted by trained clinicians as part of their clinical practice and decision-making, this is a field study (see Edens & Boccaccini, 2017). We evaluated the predictive validity of the vulnerability total score, strength total score, and the final risk judgment for inpatient adverse outcomes over a 4-month follow-up period. In addition, we explored lifetime history as a predictor; this reflects whether the adolescent has ever experienced an adverse outcome prior to the risk assessment. Incremental validity was also evaluated for multiple predictors: the strength total score over the vulnerability total scores, the total scores over lifetime history, and the final risk judgments over the total scores and lifetime history. We expected the predictive validity to be lower in our field study compared with previous nonfield studies. A field study is typically subjected to more confounding factors, such as more variability in evaluator background, training, and experience (DeMatteo et al., 2020); differences in available information (Boccaccini et al., 2008); and more time and contextual pressures (Guarnera & Murrie, 2017; Vincent et al., 2012). Confounding factors may diminish the reliability of the risk assessment ratings and, as a result, constrain predictive validity (Edens & Kelley, 2017).

## Method

The design and analysis plan were preregistered on the Open Science Framework (OSF) platform and deviations from the original plan are documented in the supplemental material, also available on the OSF page (https://osf.io/7e2hp). We report how we determined our sample size, all data exclusions, all manipulations, and all measures in the study. The study was approved by the facility’s general director and the Ethics Review Committee Psychology and Neuroscience (ERCPN) of Maastricht University (ERCPN Number 174_05_12_2016).

### Setting

The study setting is a 98-bed residential youth care service in the Netherlands with medium and high secure treatment units. On high secure units, adolescents are not allowed to leave the unit for the duration of their stay, while on a medium secure unit, adolescents can leave the unit (under supervision) to attend school, participate in leisure activities, or go on furlough. Admission to residential youth care services requires a court order by a judge who decides that a mandatory out-of-home placement in residential care is necessary to ensure the adolescent’s safety (e.g., from self-harm, abuse, and neglect) and/or the safety of their environment (e.g., violence to others, criminal behavior). This intensive type of mandated treatment is considered a “last resort” for teenage boys and girls with complex behavioral and/or mental health problems (Ten Brummelaar et al., 2017). It differs from juvenile detention because it is a civil law measure rather than a criminal sentence. In 2017, 219 adolescents (52% girls, on average 15.6 years old) resided within the service for treatment (i.e., admitted for longer than 1 month) for an average duration of 250 days (8.2 months; range = 31–853 days).

### Participants

The final sample included 42 male and 64 female adolescents between the ages of 12.4 and 18.1 years at the time of the (first) START:AV assessment (*M* = 16.1; *SD* = 1.2). Three-quarters were Dutch (82), seven Moroccan, seven Antillean, three Surinamese, three Eastern European, three Eastern African, and one Afghan. The adolescents had spent on average 97 days (3.2 months) on a secure unit until the time of data collection (*SD* = 73.53; range = 0–537) and they spent on average 345 days (11.3 months; *SD* = 134.4; range = 61–815) in the facility. Twenty-three (22%) resided on a high secure unit while the others resided on a medium secure treatment unit. The sample’s mean total IQ score was 89.4 (*SD* = 16.00; range = 54–131), assessed in 86 (81%) adolescents. IQ scores were measured using the Wechsler Intelligence Scale for Children-III-NL (WISC-III-NL; Wechsler, 2005/1991) or the Wechsler Adult Intelligence Scale-IV-NL (WAIS-IV-NL; Wechsler, 2012/2008) for adolescents of 16 and older. For the remaining 20 adolescents, a total IQ score could not be calculated because of a large discrepancy between the subscale scores (i.e., a disharmonic profile).

All youth had at least one diagnosis according to the *Diagnostic and Statistical Manual of Mental Disorders* (5th ed.; *DSM-5*; American Psychiatric Association [APA], 2013; *M =* 3.7; *SD* = 1.1; range = 1–6). The most common diagnoses were substance-related disorders (*n* = 29; 27%), oppositional defiant disorder (*n* = 26; 25%), attention-deficit hyperactivity disorder (*n* = 26; 25%), intellectual disability (*n* = 21; 20%), post-traumatic stress disorder (*n* = 20; 19%), autism spectrum disorder (*n* = 18; 17%), and conduct disorder (*n* = 16; 15%). Twenty-nine adolescents (27%) were diagnosed with an (emerging) personality disorder, mainly borderline personality disorder (24 out of 29).

### Measures

#### Short-Term Assessment of Risk and Treatability: Adolescent Version (START:AV)

The START:AV (J. L. Viljoen et al., 2014) is a risk assessment instrument that follows the structured professional judgment (SPJ) approach, meaning that the final conclusion about the level of risk is based on professional judgment rather than a statistical algorithm (Webster & Bélisle, 2014). The instrument is developed to guide an individualized assessment of future risk with the ultimate goal of preventing the occurrence of adverse outcomes (J. L. Viljoen et al., 2014). The final risk judgments for the eight adverse outcomes (Table 1) are formulated based on 26 dynamic vulnerability and strength items, as well as the adolescent’s recent and prior history of the adverse outcome. These history ratings are the only static factors in the START:AV. In the present study, we combined recent and prior history into a lifetime history variable. Lifetime history was coded as “present” when recent and/or prior history were present. In reaching a final risk judgment (*low, moderate*, or *high*), the evaluator considers two elements: (a) the likelihood that an adverse outcome will occur and/or (b) the severity of the consequences if the outcome were to occur. For adolescents who are incarcerated or in residential settings, the user guide instructs the evaluator to rate the risk “as if they were about to be discharged into the community” (J. L. Viljoen et al., 2014, p. 57). In the present study, the Dutch translation of the START:AV (J. L. Viljoen et al., 2016/2014) was used.

**Table 1.** Endorsement Frequencies for History and Final Risk Judgments of the Adverse Outcomes.

Adverse outcome	History		Final risk judgment			
	Lifetime %	Missing *n*	Low %	Moderate %	High %	Missing *n*
Violence	69.8	0	41.5	32.1	26.4	0
Nonviolent offenses	67.3	2	37.7	37.7	24.5	0
Substance abuse	72.1	2	36.2	24.8	39.0	1
Unauthorized absences	97.1	2	9.4	31.1	59.4	0
Suicide	23.8	1	84.8	12.4	2.9	1
Self-injury	39.6	0	69.2	18.3	12.5	2
Victimization	86.4	3	29.5	32.4	38.1	1
Health neglect	69.4	8	33.3	48.0	18.6	4

*Note. N* = 106.

Although START:AV strength and vulnerability total scores are not used in clinical practice, they were calculated for research purposes by summing the 26 item ratings (possible range = 0–52). For assessments with five or fewer missing items, the total score was imputed using two-way imputation (van Ginkel & van der Ark, 2005). For assessments in which medication adherence (item 23) was rated “not applicable,” the total score was prorated without item 23. START:AVs with more than five missing items were excluded (see the “Data Collection Procedure” section).

#### Inpatient Incidents

The adverse outcomes are measured with the START:AV Outcome Scale: Adolescent Version—Institutional (SOS-AVI; De Beuf et al., 2019), an adjusted version of the adult START Outcome Scale (SOS; Nicholls et al., 2007). The original SOS (Nicholls et al., 2007) is based on the Overt Aggression Scale (OAS; Yudofsky et al., 1986) supplemented with other outcomes relevant to the adult version of the START (e.g., self-harm, suicidal behaviors, self-neglect). Singh and colleagues (2014) slightly modified the SOS for use with adolescents. For the present study, we adjusted the SOS of Singh et al. (2014) to better fit the outcome descriptors in the START:AV user guide and the service’s institutional rules, and to minimize coding discrepancies. An overview of the modifications that resulted in the SOS-AVI can be accessed in the supplemental material (Table S3).

The SOS-AVI assesses nine adverse outcomes: eight START:AV outcomes, including (nonsexual) physical aggression, nonviolent offenses, unauthorized leave, self-injury, victimization, substance use, suicidal behavior, health neglect, and one additional outcome “institutional violation.” Except for suicidal behavior, incidents are coded on a 4-point severity scale, with increasing severity, based on provided indicators and coding instructions (see Appendix for an English translation of the SOS-AVI with its indicators).

The interrater reliability of the SOS-AVI was examined for 30 risk assessments and interpreted according to the guidelines of Koo and Li (2016). The two-way random, absolute agreement, single measure intraclass correlation coefficients (ICCs) were excellent for all outcomes, ranging from .91 to 1.00, except for victimization which reached good interrater reliability (ICC = .88, 95% confidence interval [CI] = [0.76, 0.94]). The interrater reliability for suicidal behavior could not be calculated because there were no suicidal behaviors coded in the cases used for the reliability check. A table with the ICCs and CIs for all SOS-AVI outcomes is available as supplemental material (Table S5).

### Data Collection Procedure

The START:AV forms were completed as part of clinical practice by 13 evaluators employed as treatment coordinators within the service. These professionals, with at least a master’s degree in psychology or special needs education, were responsible for the adolescent’s treatment. All evaluators were women, aged 26 to 40 (*M* = 32.3; *SD* = 4.5) with on average 5 years’ experience as a treatment coordinator within the organization (*SD* = 5.2; range = 0–14). They were trained by the first author who had received training and supervision from the instrument’s developers. Six (46%) had previously used other risk assessment instruments in practice and seven (54%) had received risk assessment training prior to the START:AV training. To complete the risk assessments, the evaluators had various information sources at their disposition: records (e.g., judicial, treatment, school, social service), treatment progress notes, live interactions with the adolescent and their social network, communication with other involved professionals (e.g., mentor, teacher), and, for some youth, results of formal psychological tests.

Data collection involved risk assessment forms completed between March 2016 and December 2017. During this period, 287 START:AVs were completed for clinical purposes. Eleven START:AVs were excluded because they had more than five missing strength or vulnerability ratings, 16 were excluded because they were completed by interns or temporary (untrained) employees, and finally, 68 forms were excluded because the adolescent was discharged before the end of the follow-up period. From the remaining 191 START:AVs, we randomly selected 160 cases, using SPSS’ random sampling feature. However, at the time of data-analysis, 44 cases were excluded because they were reassessments of the same youth (i.e., only the first assessment was included) or the youth was discharged during the follow-up period. The final sample comprised 106 START:AV assessments, which was sufficient for the predictive validity analyses, but did not reach the recommended sample size for correlational analyses (see the “Data Analysis” section).

For each case, a 4-month period was determined starting from the date of the risk assessment. This duration was selected because, in the present setting, the risk assessments are considered valid for a period of 4 months. In line with a prospective design, inpatient incidents that occurred within this 4-month period were coded on the SOS-AVI using daily progress notes and treatment evaluation reports available in the electronic patient files. Note that the progress notes used for coding the SOS-AVI concerned a different period (i.e., 4 months *after* the risk assessment) compared with those that were used to code the START:AV (i.e., 4 months *prior* to the risk assessment). The SOS-AVI was completed by a research assistant who was blind to the START:AV risk assessment. For each incident coded on the SOS-AVI, the date and severity were noted. The incidents were coded from 21,572 daily progress notes and 106 treatment evaluation reports. For the purpose of the present study, the SOS-AVI outcomes were dichotomized (present vs. absent) per follow-up period.

### Data Analysis

#### Sample Size Calculation

Preliminary research on the predictive validity of the START:AV (J. L. Viljoen, Beneteau, et al., 2012) found significant area under the curve (AUC) values ranging from .69 to .82. To detect these AUCs with a power of .80 and α of .05, a minimum sample size of 70 is required (MedCalc version 16.2.0, 2016). Using the lowest of the significant correlations in the study of Jodi Viljoen, Beneteau, et al. (*r* =|.23 − .51|; 2012), with a power of .80 at a two-tailed α of .05, a sample size of 143 is required (Faul et al., 2009).

#### Descriptive and Correlational Analyses

Descriptive statistics were calculated for the START:AV items, total scores, and adverse outcomes, as well as for the SOS-AVI outcomes. Correlations were calculated between the START:AV total scores and final risk judgments (Kendall’s *τ*_b_), as well as between total scores and SOS-AVI outcomes (point-biserial). The association of the final risk judgments with the history ratings as well as with the SOS-AVI outcomes was measured using the chi-square statistic and the strength of association was reported using Cramer’s *V* or Phi. Correlation coefficients of .10, .30, and .50 are typically considered small, moderate, and large, respectively (Cohen, 1992). Note that all CIs in this paper are 95% CIs.

#### Interrater Reliability

Interrater reliability for the sample was evaluated using ratings of the treatment coordinators compared with those of research assistants (as second evaluators). The research evaluators based their ratings on file information only, whereas the treatment coordinators additionally relied on their own observations and interactions with the adolescent and the team, as described earlier. Two-way random ICCs, single measure, were calculated for the total scores (consistency type) and the final risk judgments (absolute agreement) as well as Gwet’s agreement coefficient (AC) for lifetime history, based on 30 randomly selected cases (28%). Gwet’s AC for dichotomous variables is preferred to other coefficients because it remains stable with varying prevalence rates (Vial et al., 2019). Other coefficients, such as kappa and Krippendorff, tend to underestimate agreement in situations with high or low prevalence (e.g., prevalence of suicide), a phenomenon referred to as the “kappa paradox” or the “paradox of high agreement, low reliability” (Feinstein & Cicchetti, 1990). Gwet’s AC was calculated using the AgreeStat360 Excel program (Gwet, 2020). The ICC values and Gwet’s ACs were interpreted according to Koo and Li’s (2016) guidelines: ICC < .50, poor; .50 to .75, moderate; .75 to .90, good; > .90, excellent. We used these stricter benchmarks in line with the argument that classic benchmarks, such as those by Landis and Koch (1977), Cicchetti and Sparrow (1981), or Fleiss (1986), might be too lenient for applied settings (Edens & Boccaccini, 2017; Levenson, 2004). Life-altering decisions are made based on the findings from risk assessments; therefore, lower tolerance of error is warranted.

#### Predictive Validity and Incremental Validity

The total scores, history ratings, and final risk judgments were included as independent variables in the predictive validity analyses. The nine SOS-AVI outcomes, rated as present versus absent, were the dependent variables. Predictive validity of risk assessment instruments is most commonly measured using a receiver operating characteristic (ROC) curve analysis which plots the true-positive rate (sensitivity) against the false-positive rate (1 − specificity) for every possible cut-off threshold (Singh, 2013). The resulting AUC values are indicators of discrimination that indicate how well the instrument can discriminate between people who experienced adverse outcomes versus those who did not experience adverse outcomes (Singh, 2013). More specifically, an AUC of .50 indicates a classification no better than chance, while an AUC of 1.00 indicates 100% accurate classification. This performance indicator is recommended for examining predictive validity because it is resistant to fluctuating base rates and independent of cut-off thresholds (Singh, 2013). For each AUC value, we also report the approximate Cohen’s *d* based on calculations by Rice and Harris (2005; see Table, p. 616), with *d* values of .20, .50, and .80 representing a small, medium, and large effect size (Rice & Harris, 2005).

ROC curve analysis is not possible for dichotomous independent variables, such as the history ratings (present/absent); therefore, a binominal logistic regression was used to assess their predictive validity. To analyze the incremental validity of the final risk judgments over the vulnerability and strength total scores, as well as the incremental validity of the vulnerability and strength total scores over the lifetime history ratings, hierarchical (block-entry) binominal logistic regressions were conducted. For hierarchical logistic regressions, there is a sample size requirement referred to as the “events per variable” (EPV) rule. This rule, established to prevent model overfitting, informs about the maximum number of predictors in a model based on the number of events in the smallest of the outcome’s categories. The EPV rule is typically set at 10 events per predictor (Peduzzi et al., 1996).

In logistic regression, nonbinary categorical variables such as the final risk judgments are dichotomized by creating dummy variables that are compared to a reference category. We selected the absence-category as the reference category for the history ratings and the low-risk category for the final risk judgments. The index of interest in logistic regressions is the odds ratio (OR): the ratio of the odds of an incident occurring in the group identified as high (or moderate) risk compared to the odds of an incident occurring in the group identified as low risk. An OR of 1 indicates that there is no difference, an OR greater than 1 means a positive association with the outcome, whereas an OR less than 1 indicates a negative association with the outcome (i.e., the odds of an adverse outcome occurring are lower).

In exploratory post hoc analyses, we calculated the correlation between ICC values and AUC values for the final risk judgments, and we added “alcohol-soft drugs” and “hard drugs” as separate outcomes in validity analyses for reasons of comparison with previous studies (e.g., J. L. Viljoen, Beneteau, et al., 2012). All analyses in this study (except the calculation of Gwet’s AC) were conducted using IBM SPSS Statistics 27.

## Results

### Descriptive and Correlational Analyses of the START:AV Risk Assessments

The mean total score of the 106 risk assessments was 18.66 for strengths (*SD* = 8.36, range = 3–45) and 31.73 for vulnerabilities (*SD* = 6.26, range = 19–47). The strength and vulnerability total scores had a moderate correlation in the expected direction (*r* = −.48, *p* < .001, CI = [−0.62, −0.32]). Means and standard deviations for the START:AV items are available as supplemental material on the OSF page (Table S6). As shown in Table 1, lifetime history was rated as present in 24% (suicide attempts) up to 97% (unauthorized absences) of the assessments. The table also displays the distribution of the final risk judgments for each adverse outcome. The low-risk category was most commonly selected for violence, suicide, and self-injury, and the high-risk category was most frequently endorsed for substance abuse, unauthorized absences, and victimization.

Furthermore, both total scores were significantly correlated with the final risk judgments for violence, nonviolent offenses, unauthorized absences, and health neglect (Table 2). Lifetime history was significantly and strongly correlated with the final risk judgments for all adverse outcomes except unauthorized absences.

**Table 2.** Correlations of Final Risk Judgments with Total Scores and Lifetime History.

Final risk judgment	N1	Strength total	Vulnerability total	N2	Lifetime history
Violence	106	−.41***	.23*	106	.78***
Nonviolent offenses	106	−.32**	.24*	104	.80***
Substance abuse	105	.01	.07	103	.69***
Unauthorized absences	106	−.29**	.30**	104	.23
Suicide	105	.10	.00	104	.58***
Self-injury	104	−.01	.07	104	.78***
Victimization	105	−.02	.16	102	.52***
Health neglect	102	−.29**	.40***	96	.81***

*Note.* N1 refers to the sample for the correlations with the total scores, whereas N2 refers to the correlations with lifetime history.

* *p* < .05. **p < .01. ***p < .001.

### Descriptive Analyses of Inpatient Incidents as Adverse Outcomes

From the 21,572 progress notes that were studied, 3,065 incidents were coded. All youth in the present sample, except one, had incidents in the 4 months after their risk assessment. Table 3 describes the prevalence of adverse outcomes in the sample as well as the descriptive statistics for the individual incidents per outcome. The prevalence of institutional violations, unauthorized leave, and nonviolent offenses was high, with a large majority (>80%) of adolescents demonstrating these adverse outcomes. Physical aggression, victimization, and substance use were also relatively common in this sample. In addition, some form of health neglect and self-injury was observed in almost half of the follow-up periods. The prevalence of suicide attempts was low; therefore, this outcome was excluded from further analyses. For most outcomes, the majority of incidents occurred in the low severity categories (see supplemental material Table S7).

**Table 3.** Prevalence of SOS-AVI Outcomes and Unique Incidents During Follow-Up.

SOS-AVI outcome	Presence		Incidents			
	*n*	%	*M*	*SD*	Range	Total
Physical aggression	78	73.6	3.85	4.89	0–26	408
Nonviolent offenses	88	83.0	5.28	5.29	0–24	560
Institutional violation	98	92.5	8.47	7.82	0–43	898
Substance use	59	55.7	2.37	3.33	0–15	251
Unauthorized leave	91	85.8	4.14	4.58	0–28	439
Suicidal behavior	4	3.8	0.05	0.25	0–2	5
Self-injury	44	41.5	2.05	4.44	0–30	217
Victimization	67	63.2	2.26	3.16	0–15	240
Health neglect^a^	47	44.3				47
Any incidents	105	99.1	28.92	19.14	0–91	3,065

*Note. N* = 106.

a Health neglect was not counted, but rated as present/absent over the course of 4 months.

### Interrater Reliability

As shown in Table 4, the interrater reliability for the total scores was weak and poor to good for lifetime history and final risk judgments. Gwet’s ACs for lifetime history ranged from .44 for health neglect to .92 for unauthorized absences. The ICCs (absolute agreement) for the final risk judgments ranged from .46 for health neglect to .86 for suicide. This reliability sample was part of a larger field interrater reliability evaluation of the START:AV (De Beuf et al., 2021).

**Table 4.** Interrater Reliability of Lifetime History and Final Risk Judgments, and Their Interpretation According to Koo and Li (2016).

Adverse outcome	*n*	IRR	IRR interpretation	95% CI	CI interpretation
Vulnerability Total Score	30	.46	Poor	[0.13, 0.70]	Poor–Moderate
Strength Total Score	30	.29	Poor	[–0.08, 0.58]	Poor–Moderate
Lifetime history					
Violence	30	.63	Moderate	[0.33, 0.92]	Poor–Excellent
Nonviolent offenses	29	.84	Good	[0.65, 1.00]	Moderate–Excellent
Substance abuse	29	.72	Moderate	[0.46, 0.98]	Poor–Excellent
Unauthorized absences	30	.92	Excellent	[0.80, 1.00]	Good–Excellent
Suicide	30	.78	Good	[0.56, 1.00]	Moderate–Excellent
Self-injury	30	.62	Moderate	[0.32, 0.91]	Poor–Excellent
Victimization	30	.82	Good	[0.62, 1.00]	Moderate–Excellent
Health neglect	28	.44	Poor	[0.08, 0.81]	Poor–Good
Final risk judgment					
Violence	30	.62	Moderate	[0.34, 0.80]	Poor–Good
Nonviolent offenses	30	.74	Moderate	[0.51, 0.87]	Moderate–Good
Substance abuse	30	.80	Good	[0.63, 0.90]	Moderate–Good
Unauthorized absences	30	.57	Moderate	[0.27, 0.77]	Poor–Good
Suicide	30	.86	Good	[0.72, 0.93]	Moderate–Excellent
Self-injury	30	.83	Good	[0.67, 0.92]	Moderate–Excellent
Victimization	30	.62	Moderate	[0.22, 0.82]	Poor–Good
Health neglect	30	.46	Poor	[0.13, 0.70]	Poor–Moderate

*Note.* Reliability is calculated using Gwet’s accuracy coefficient for lifetime history and the intraclass correlation coefficient for the final risk judgments. CI = confidence interval; IRR = interrater reliability.

### Predictive Validity

#### Vulnerability Total Score

As shown in Table 5, the vulnerability total score was significantly correlated with only two adverse outcomes: nonviolent offenses and institutional violations. However, when assessed with the ROC curve, the vulnerability total score was only predictive of institutional violations, demonstrating a large effect size. The ORs indicated that a 1-point increase on the vulnerability total score resulted in the odds of violating institutional rules being 1.29 times greater (OR = 1.29, CI = [1.09, 1.53], *p* = .004).

**Table 5.** Correlations and Predictive Validity Parameters for the Vulnerability Total Score per SOS-AVI Outcome.

SOS-AVI outcome	*r*	*p*	AUC	*SE* of AUC	95% CI	*p*	Cohen’s *d*
Physical aggression	.17	.086	.61	0.065	[0.48, .073]	.099	0.40
Nonviolent offenses	.19	.046	.64	0.078	[0.48, 0.79]	.067	0.50
Institutional violation	.32	.001	.82	0.085	[0.66, 0.99]	.003	1.33
Substance use	.07	.475	.53	0.057	[0.42, 0.64]	.620	0.11
Alcohol-soft drugs	.11	.267	.55	0.056	[0.44, 0.66]	.376	0.18
Hard drugs	.03	.773	.52	0.071	[0.38, 0.66]	.819	0.07
Unauthorized leave	.08	.427	.58	0.082	[0.42, 0.75]	.301	0.29
Self-injury	−.03	.770	.48	0.058	[0.37, 0.60]	.758	0.00
Victimization	−.05	.642	.48	0.058	[0.36, 0.59]	.685	0.00
Health neglect	.05	.615	.54	0.056	[0.43, 0.65]	.506	0.14

*Note. N* = 106. SOS-AVI = START:AV Outcome Scale: Adolescent Version—Institutional; AUC = area under the curve; SE = standard error; CI = confidence interval.

#### Strength Total Score

The strength total score was also significantly related to and predictive of institutional violations, with a large effect size (Table 6). The strength total score also predicted physical aggression, demonstrating a small effect. When calculating the ORs for these outcomes, we found that a 1-point increase on the strength total score resulted in 1.06 greater odds of *not* being physically aggressive (OR = 1.06, CI = [1.00, 1.12], *p* = .050) and 1.10 greater odds of *not* violating institutional rules (OR = 1.10, CI = [1.01, 1.20], *p* = .038).

**Table 6.** Correlations and Predictive Validity Parameters for the Strength Total Score per SOS-AVI Outcome.

SOS-AVI outcome	*r*	*p*	AUC	*SE* of AUC	95% CI	*p*	Cohen’s *d*
Physical aggression	−.19	.046	.63	0.065	[0.50, 0.76]	.039	.47
Nonviolent offenses	−.16	.099	.63	0.093	[0.44, 0.81]	.096	.47
Institutional violation	−.21	.029	.73	0.105	[0.52, 0.93]	.033	.86
Substance use	−.02	.807	.47	0.057	[0.36, 0.58]	.589	.00
Alcohol-soft drugs	−.04	.720	.49	0.057	[0.38, 0.60]	.897	.00
Hard drugs	−.12	.219	.40	0.079	[0.25, 0.56]	.249	.00
Unauthorized leave	−.04	.680	.55	0.090	[0.38, 0.73]	.535	.18
Self-injury	.13	.182	.44	0.056	[0.33, 0.55]	.283	.00
Victimization	.01	.956	.52	0.057	[0.40, 0.63]	.793	.07
Health neglect	.06	.512	.47	0.057	[0.36, 0.58]	.620	.00

*Note. N* = 106. For interpretation purposes, AUC values for Strength Total scores were analyzed to predict the *absence* of outcomes. SOS-AVI = START:AV: AV Outcome Scale: Adolescent Version—Institutional; AUC = area under the curve; SE = standard error; CI = confidence interval.

Next, we were interested in the incremental validity of the strength total score over the vulnerability total score. However, there were not enough EPV for institutional violations to conduct a hierarchical logistic regression with two predictors (Table 3).

#### History

Correlational and predictive analyses of lifetime history were significant for at least six adverse outcomes (Table 7). No significant (predictive) associations were found between lifetime history and nonviolent offenses, unauthorized leave, and health neglect. The logistic regression analysis for hard drug use could not be conducted because one cell in the contingency table had zero observations, resulting in insufficient information to calculate the OR. The significant ORs ranged from 3.92 for victimization to 10.20 for alcohol and soft drug use. We found no incremental validity of the strength total score over lifetime history for physical aggression, ∆χ^2^(1) = 1.67, *p* = .196. Due to insufficient EPV, incremental validity of the total scores over lifetime history could not be conducted for institutional violations.

**Table 7.** Correlations and Predictive Validity Parameters for Lifetime History per SOS-AVI Outcome.

SOS-AVI outcome	*n*	Φ	*p*	*B (SE)*	Wald χ^2^	*p*	OR	95% CI
Physical aggression	106	.35	.000	1.64 (0.47)	12.02	.001	5.17	[2.04, 13.08]
Nonviolent offenses	104	−.09	.379	−0.54 (0.62)	0.76	.382	0.59	[0.18, 1.95]
Institutional violation	104	.26	.008	1.99 (0.85)	5.50	.019	7.29	[1.39, 38.31]
Substance use	104	.44	.000	2.16 (0.52)	17.09	.000	8.67	[3.11, 24.13]
Alcohol-soft drugs	104	.46	.000	2.32 (0.55)	17.80	.000	10.20	[3.47, 30.00]
Hard drugs^a^	104	.25	.012	—	—	—	—	—
Unauthorized leave^a^	104	−.07	.471	—	—	—	—	—
Self-injury	106	.37	.000	1.61 (0.43)	14.01	.000	4.98	[2.15, 11.53]
Victimization	103	.23	.017	1.37 (0.60)	5.14	.023	3.92	[1.20, 12.78]
Health neglect	98	.08	.435	0.35 (0.45)	0.61	.436	1.41	[0.59, 3.38]

*Note.* SOS-AVI = START:AV: AV Outcome Scale: Adolescent Version—Institutional; Φ = Phi; SE = standard error; OR = odds ratio; CI = confidence interval.

a Zero observations in one of the cells of the contingency table prevented further analysis.

#### Final Risk Judgments

All final risk judgments had significant correlations with their respective outcome, except unauthorized leave and health neglect (Table 8). Likewise, the final risk judgments were significant predictors of most outcomes, with statistically significant AUCs for physical aggression, institutional violation, substance use, self-injury, and victimization. The significant AUC values ranged from .62 to .80. The AUCs of institutional violations, hard drug use, and alcohol-soft drug use represented a large effect size, the AUCs of physical aggression, overall substance use, and self-injury a moderate effect, and we found a small effect for victimization.

**Table 8.** Correlations and Predictive Validity Parameters for the Final Risk Judgments per SOS-AVI Outcome.

SOS-AVI outcome	Final risk judgments									
	*N*	φ_*c*_	*p*	AUC	*SE* of AUC	95% CI	*p*	Cohen’s *d*	OR moderate vs. low [CI]	OR high vs. low [CI]
Physical aggression	106	.34	.002	.71	0.054	[0.60, 0.82]	.001	0.78	2.93 [1.05, 8.16]	9.88 [2.08, 46.88]
Nonviolent offenses	106	.26	.025	.62	0.061	[0.50, 0.74]	.103	0.43	0.75 [0.26, 2.15]	^a^
Institutional violation	106	.30	.010	.79	0.063	[0.66, 0.91]	.007	1.15	^b^	^b^
Substance use	105	.42	.000	.71	0.052	[0.61, 0.81]	.000	0.78	5.52 [1.86, 16.40]	6.69 [2.50, 17.91]
Alcohol-soft drugs	105	.42	.000	.72	0.051	[0.62, 0.82]	.000	0.82	4.48 [1.54, 13.07]	7.64 [2.81, 20.74]
Hard drugs	105	.39	.000	.80	0.048	[0.71, 0.89]	.000	1.19	^a^	^a^
Unauthorized leave	106	.12	.495	.42	0.077	[0.27, 0.57]	.323	0.00	1.11 [0.10, 12.04]	0.53 [0.06, 4.58]
Self-injury	104	.39	.000	.68	0.055	[0.57, 0.79]	.002	0.66	3.13 [1.11, 8.84]	12.50 [2.55, 61.17]
Victimization	105	.25	.036	.62	0.058	[0.51, 0.74]	.035	0.43	2.91 [1.05, 8.10]	3.20 [1.19, 8.62]
Health neglect	102	.15	.297	.52	0.057	[0.40, 0.63]	.770	0.07	1.83 [0.75, 4.45]	0.94 [0.30, 3.01]

*Note. N* = 106. SOS-AVI = START:AV: AV Outcome Scale: Adolescent Version—Institutional; φ_*c*_ = Cramer’s *V*; AUC = area under the curve; SE = standard error; CI = confidence interval; OR = odds ratio.

a Zero observations in one of the cells of the contingency table prevented further analysis. ^b^Not enough events per variable to conduct binominal regression analysis.

The incremental validity of the final risk judgment over the total scores could not be calculated, because the outcomes (i.e., physical aggression, nonviolent offenses, and institutional violations) did not have enough EPVs for a model with three predictors (i.e., one total score and two dummy variables for the final risk judgment). For the analysis of incremental validity of the final risk judgment over lifetime history, there were enough EPVs for substance use, self-injury, and victimization. For none of these adverse outcomes, the final risk judgment added significant incremental validity over lifetime history. Despite not finding significant change indices, the model with lifetime history and the final risk judgment was statistically significant, explaining 28% of the variance in substance use, 22% of the variance in self-injury, and 10% of the variance in victimization (for details, see supplemental material Table S8).

#### Post Hoc Correlation Between Interrater Reliability and Validity

A post hoc correlational analysis between the interrater reliability values (ICC) and predictive validity values (AUC) of the final risk judgments (*N* = 8) yielded a large nonsignificant association, *r* = .67, *p* = .071.

## Discussion

The present field study evaluated the START:AV’s predictive accuracy for the occurrence of adverse outcomes within medium and high secure residential youth care, using a short-term prospective design. The START:AV assessments were conducted by trained professionals as part of clinical decision-making with real-life implications. Therefore, this study adds to the literature on field validity of risk assessment instruments. Furthermore, to the best of our knowledge, this study is the first to assess the predictive validity of the final version of the START:AV and more specifically, its Dutch translation.

In the present study, the START:AV total scores demonstrated limited predictive validity. Both total scores predicted institutional violations, an adverse outcome specifically constructed for the present context, and the strength total score additionally predicted physical aggression. The latter finding is in line with previous START:AV validity studies, as is the lack of predictive validity for self-injury (Sher et al., 2017; J. L. Viljoen, Benetaeu, et al., 2012; J. L. Viljoen, Shaffer, et al., 2015; S. Viljoen, 2014). However, unlike previous studies, the total scores were not predictive of nonviolent offenses, substance use, unauthorized absences, victimization, and health neglect. Although we had expected to find lower AUC values (due to lower field reliability), these nonsignificant results were surprising. For example, in another START:AV field study, Sher and colleagues (2017) found that both total scores were significant predictors of nonviolent offenses, operationalized as property aggression, and the vulnerability total score was a significant predictor of physical aggression. One possible explanation for this difference is that their risk assessments may have been more accurate, because they were conducted by a multidisciplinary team rather than by a single evaluator. This consensus-based approach has previously shown to result in the highest predictive accuracy (de Vogel & de Ruiter, 2006).

Alternatively, the absence of significant predictive accuracy for the total scores may stem from the multiple adverse outcomes approach of the START:AV. In line with this, Braithwaite and colleagues (2010) hypothesized for the adult START that total scores may be nonsignificant predictors because not all items included in the total score are relevant for every adverse outcome. To explore this hypothesis in a civil psychiatric sample, the authors developed what they labeled “optimized vulnerability and strength scales” by including only the items that demonstrated a significant association (*p* < .05) with the START outcome of interest. They demonstrated that the optimized total scores, compared to the original total scores, predicted incidents of suicidality, substance use, self-neglect, and victimization significantly better. Applying this to the START:AV, which promotes the SPJ approach, we hypothesize that merely summing the item scores is not predictive of every outcome in the present sample, and more selectively composed total scores may improve accuracy. Moreover, the predictive validity of the total scores is less clinically meaningful, because such actuarial use is not recommended for SPJ instruments. The final risk judgments are considered to be the most meaningful predictors in the SPJ approach (Heilbrun et al., 2021). It is more relevant for future research to examine the importance of individual vulnerability and strength items for each adverse outcome within a particular target group. This knowledge may then inform professionals on how to weigh and integrate items when making a final risk judgment for each adverse outcome.

Lifetime history of an adverse outcome was highly predictive of experiencing the same outcome in the short-term, except for nonviolent offenses, unauthorized leave, and health neglect. For the majority of adverse outcomes, having experienced the outcome in the past considerably increased the odds of re-experiencing the outcome in the short term: up to 10 times. We found strong associations between history ratings and the final risk judgments, which suggests that evaluators relied heavily on historical information to formulate a final risk judgment. Although this helps to identify youth at risk, historical information is less useful for risk management because it cannot be targeted for risk reduction (Douglas & Kropp, 2002). Dynamic factors, on the contrary, are key to risk management and the assessment of changes in risk level. Sellers et al. (2017) demonstrated that START:AV assessments could detect changes in strengths and vulnerabilities in a residential juvenile justice sample over a 3-month follow-up. In addition to research on the validity of the individual strength and vulnerability items, future studies should examine whether dynamic change improves risk prediction (J. L. Viljoen et al., 2017).

The majority of final risk judgments were predictive of their respective outcomes, including physical aggression, institutional violations, substance use, self-injury, and victimization. In line with previous research, the final risk judgment was not predictive of unauthorized leave and health neglect; however, in our study, it was also not predictive of nonviolent offenses. In previous START:AV studies, the findings for nonviolent offenses varied depending on the operationalization of the outcome: Simone S. Viljoen (2014) found that the final risk judgment was a significant predictor of property damage, whereas Jodi J. L. Viljoen and colleagues (2012) did not find a significant association with arrests for any offense. Whereas the first operationalization is a narrow definition of the outcome, the latter is too broad because the authors also included violent offenses. In the present study, the outcome was operationalized in line with the definition in the START:AV user guide, including behaviors ranging from disorderly conduct, to vandalism and drug possession, to burglary and selling drugs. The differences in outcome measurement impede comparison between studies.

The predictive validity of the final risk judgments for unauthorized absences and health neglect may have been affected by their limited interrater reliability. Given the rather strong correlation between ICC and AUC values (*r* = .67), lower reliability may have affected validity, especially for these two final risk judgments, which had the lowest ICC values in our sample. We note that the correlation between the ICC and AUC values was not significant, most likely because of the small sample on which this correlation was calculated (i.e., eight final risk judgments). That said, the inability of the final risk judgment to predict unauthorized absences and health neglect was also found in S. Viljoen’s study (2014) as well as in studies with the adult START (O’Shea & Dickens, 2014, 2016). Future research should investigate whether this is due to difficulties in the conceptualization of these adverse outcomes, the inability of the START:AV to predict these outcomes, or whether it reflects measurement issues of the outcome scale.

### Incremental Validity

We found no evidence for incremental validity of the strengths over the vulnerabilities for institutional violations. This is in line with J. L. Viljoen, Beneteau, and colleagues (2012) who failed to find incremental validity of the strength total score over the vulnerability score for other adverse outcomes, such as self-reported street drug use, violence, and offending. We asked ourselves whether the strengths and vulnerabilities in the START:AV are distinct enough to represent discrete features and thus explain additional variance. We believe they are separate constructs, as supported by the limited correlation between the strength and vulnerability total scores (*r* = −.48). The correlations for the strengths and vulnerabilities of individual items (see supplemental material Table S6) were significant and moderate in size (i.e., the largest correlation was −.52, for substance use). Desmarais et al. (2012) found similar associations between both sides of the items but a smaller correlation between the total strength and vulnerability scores (*r* = −.22). Comparable to the present study, their START:AV assessments were conducted within a residential setting (i.e., juvenile detention). Studies with community probation samples found larger correlations between strengths and vulnerabilities. For instance, J. L. Viljoen, Beneteau, et al. (2012) found a correlation of −.74 between the strength and vulnerability total score, and Klimukienė et al. (2018) reported a correlation of −.76. This could mean the anchors of the START:AV strengths and vulnerabilities represent separate constructs, perhaps depending on the context and the availability of information. It may be interesting for future studies to examine whether strengths with smaller associations with their vulnerability counterparts demonstrate higher incremental validity over vulnerability. This would confirm the capacity of strengths to add new information that improves risk prediction. Furthermore, it would be relevant to examine incremental validity with optimized total scores rather than the original total scores.

Next, we found no incremental validity of final risk judgments over lifetime history, suggesting that the formulation of a risk estimate of low, moderate, or high risk in this sample did not add predictive value beyond lifetime incidents. To our knowledge, this has not been previously examined in START:AV studies. When consulting research on the START adult version, we found one study that reported incremental validity of the suicide final risk judgment over lifetime history of suicide attempts for the prediction of self-harm among forensic psychiatric inpatients (Lam, 2014). In another START study, O’Shea and Dickens (2016) found that the final risk judgment added incremental predictive validity over the total scores, lifetime history, and recent history (i.e., previous 3 months) for victimization and a combined self-harm/suicide outcome, but not for aggression and self-neglect. Our finding that the final risk judgments did not explain additional variance in substance use, self-injury, and victimization—although they were significant predictors—may suggest that the evaluators relied too much on historical information when formulating a final risk estimate. Indeed, these final risk judgments correlated strongly with the ratings of lifetime history, but not with the total scores (Table 2). This finding taps into the call for more in-depth examination of the human decision-making process in forensic risk evaluations (Guarnera & Murrie, 2017), in this case, how evaluators reach a final risk judgment.

### Limitations

As mentioned earlier, a first limitation is the relatively low interrater reliability for the total scores and several final risk judgments. These reliability ratings represent the agreement between clinicians and researchers who rated the same cases, however, with differences in the information that was available for each evaluator group (De Beuf et al., 2021). This may reflect real-world differences between evaluators in the field and, therefore, provide insight in the actual field performance of the risk assessment instrument and the impact on predictive validity. Second, based on the a priori power analysis described in the preregistration, our final sample size was too small for the correlational analyses. This may have limited the chances of finding significant correlations, for example, for the total scores. Nevertheless, the study was sufficiently powered to assess predictive validity. Third, because of the high base rates in our sample, we were unable to perform most hierarchical logistic regression analyses. The high base rates were likely the result of the data collection process: by scrutinizing daily progress notes, many minor incidents were detected, including ones that would arguably not be identified when relying on self-report, staff report, or official records. This should be taken into consideration when comparing the present study with others. Despite the high base rates, the START:AV demonstrated predictive validity for multiple adverse outcomes. Fourth, it should be noted that the use of inpatient incidents as the dependent variable is not entirely in line with the objective of the final risk judgments. According to the START:AV user guide, the final risk judgments within a residential setting should be rated as if the adolescent is no longer residing within a secure, supervised environment. This instruction was applied to the current setting; thus, clinicians were making predictions about future risk in the community rather than a residential setting. However, the majority of the adolescents in our sample resided on medium secure units, which allowed them to practice with liberties and responsibilities, including going on leave in the community.

### Implications for Research and Practice

In addition to the suggestions for future research mentioned throughout the “Discussion” section, an important next step is the replication of the predictive validity of the START:AV for this population using a larger sample and for various subgroups, for instance, based on gender, age, psychopathology, or supervision level (medium vs. high secure). Future research should evaluate whether the START:AV is equally valid for boys and girls in secure youth care settings, as was previously observed for adolescents in probation services (J. L. Viljoen, Beneteau, et al., 2012). Future research needs to examine the predictive validity of individual strength and vulnerability items and, perhaps most importantly which items (or change in items) predict which adverse outcome. Understanding which items explain additional variance will be useful to inform intervention strategies. Furthermore, it would be interesting for future research to evaluate the predictive validity for frequency and severity of incidents, in addition to mere presence or absence. In addition to evaluating the field validity of the START:AV in similar and other settings, it is important to evaluate the relevance of the instrument to intervention planning. Does use of the START:AV affect risk management outcomes, such as a reduction in incidents or a decrease in liberty-restricting measures? As with other risk assessment instruments that inform risk management, more research is needed into whether the START:AV can effectively guide practitioners on the path from risk assessment to risk management (see also J. L. Viljoen & Vincent, 2020).

With its focus on dynamic factors, the START:AV is, at face value, well-suited for risk assessment within a specialized youth care service that treats adolescents with complex problems. Indeed, the present study empirically demonstrated the relevance of the START:AV final risk judgments, formulated by trained clinicians, for the prediction of multiple inpatient adverse outcomes in a secure youth care setting over a 4-month follow-up. This supports the utility of the START:AV to guide treatment planning and decision-making (e.g., regarding furlough or discharge). Based on the current findings, caution is advised when using the START:AV for the prediction of nonviolent offenses, unauthorized absences, and health neglect in a secure residential setting. Furthermore, we found that clinicians in the present setting relied considerably on past occurrences of adverse outcomes to reach a final risk judgment. This finding is at odds with the general view of adolescence as a highly dynamic developmental phase. It is essential for evaluators to consider dynamic risk and protective factors in risk assessment and intervention planning. This should be emphasized during training and rehearsed when applying the START:AV.

## Conclusion

The present study demonstrated the short-term predictive validity of the START:AV final risk judgments for inpatient incidents in a Dutch residential youth care setting that serves a complex population of adolescents with severe behavioral problems and mental health issues, often in combination with serious interpersonal problems (e.g., complicated parent–child interactions). This was the first study on the validity of the START:AV that used the final version of the user guide, specifically the Dutch translation. It adds to the available research on field validity of the START:AV by demonstrating the validity of clinician-rated final risk judgments for adverse outcomes beyond violence. Additional field studies with different adolescent samples are warranted to further establish in which target groups the START:AV can be applied effectively.

## Supplemental Material

sj-docx-1-asm-10.1177_10731911211063228 – Supplemental material for Prospective Field Validation of the START:AV in a Dutch Secure Youth Care Sample Click here for additional data file. Supplemental material, sj-docx-1-asm-10.1177_10731911211063228 for Prospective Field Validation of the START:AV in a Dutch Secure Youth Care Sample by Tamara L. F. De Beuf, Vivienne de Vogel, Nick J. Broers and Corine de Ruiter in Assessment

## Appendix

**Appendix.** START:AV Outcome Scale: Institutional (SOS-AVI; De Beuf et al., 2019).

START:AV CODE:		SOS RATER:	
SOS TIME PERIOD:		SECURITY LEVEL: HIGH/MEDIUM/LOW	
☐ Adolescent moved to unit with another security level on __ - __ - ____ to HIGH/MEDIUM/LOW			
☐ Adolescent discharged prior to the end of the SOS period. Discharge date: __ - __ -___			
Mark each severity level per incident that was present during the 17-week period after the START:AV completion date. Record the date and source of each event. Consult the additional coding guidelines for detailed instructions.			
**[1]**	**Physical Aggression** (nonsexual)	**[2]**	**Institutional Violation**
DATE (SOURCE)	☐ NO	DATE (SOURCE)	☐ NO
	1. Makes threatening gestures/has threatening posture/threatens to physically harm someone, swings at people, grabs at clothing, deliberately spits on people.		1. Lending/borrowing/trading items or clothing; refusing to adhere to the dress code.
	2. Physical aggression not resulting in injury, such as hitting, pushing, scratching, pulling hair. Throws object toward others without injuring the other.		2. Possession of contraband (other than drugs and weapons; for example, phone, lighter). Without permission of staff in one another’s room. Aids or abets other youth in any level 2 infraction.
	3. Acts that (potentially) result in mild to moderate physical injury (e.g., bruises, sprain, welts), such as kicking, punching, biting. Throws object directed at what (potentially) results in minor injury.		3. Enter another youth’s room without his or her permission. Present in places where one is not allowed. Smoking without permission/on prohibited time or location. Tattooing or body piercing self or others. Consensual sexual touching/fondling on the premises. Aids or abets other youth in any level 3 infraction.
	4. Acts that (potentially) result in serious physical injury (e.g., fracture, loss of teeth or consciousness, lacerations, internal injury), such as attacking others, using weapons toward others. Throws object directed at others what (potentially) results in serious injury.		4. Organizes resistance against staff, tampers with safety equipment (smoke detector, fire doors, alarm). Consensual sexual intercourse on the premises. Aids or abets other youth in any level 4 infraction. Assists in absconding of another youth.
	99. Severity not described/provided		99. Severity not described/provided.
**[3]**	**Nonviolent Offenses**	**[4]**	**Unauthorized Leave**
DATE (SOURCE)	☐ NO	DATE (SOURCE)	☐ NO
	1. Disorderly conduct (e.g., hooliganism, noise pollution, throwing objects [nondirected]). Destroys own property. Illegally paint graffiti. Fare-dodging.		1. Returns late from unescorted leave without prior notification or adequate explanation. Arrives late at leave address without valid reason. Absent from school without valid reason. Arrives deliberately late at therapy or mandatory group activity, or stops earlier without permission.
	2. In possession of soft drugs. Trespasses. Steals objects of limited value. Commits vandalism. Aids or abets other youth in any level 2 offense.		2. Returns from unescorted leave 24 hours or more late. Plays truant for multiple straight. Missed therapy appointment without valid reason.
	3. In possession of hard drugs. Gambles. Hacks. Aids or abets other youth in any level 3 offense.		3. Absconds from escorted leave or is returned by police from unescorted leave or does not return. Is absent from school for longer than 4 weeks. Refuses to go to therapy.
	4. Commits burglary. Sells drugs. In possession of weapons. Steals valuables or money (e.g., from staff). Extorts. Aids or abets other youth in any level 3 offense.		4. Escapes from secure setting. Stops with school or is not registered in a school. Stops (prematurely) with therapy (one-side decision).
	99. Severity not described/provided		99. Severity not described/provided.
**[5]**	**Self-Injury**	**[6]**	**Victimization**
DATE (SOURCE)	☐ NO	DATE (SOURCE)	☐ NO
	1. Picks or scratches skin, pulls out hair, hits self (without injury).		1. Bullied or intimidated by others, resulting in mild emotional harm, financial/material harm or mild fear and intimidation.
	2. Bangs head, hits fist into objects, throws self onto floor or into objects (acts resulting in minor injury).		2. Abused or verbally threatened, resulting in moderate to severe emotional harm, intimidation, fear, financial/material harm, but without physical injury.
	3. Self-mutilation resulting in moderate injuries (small cuts or bruises, minor burns).		3. Physically assaulted resulting in mild to moderate physical injury (e.g., bruises, sprains, or welts) or nonconsensual sexual touching or fondling.
	4. Mutilates self, makes deep cuts, internal injury, fracture, loss of consciousness, loss of teeth. Self-harm (potentially) resulting in hospitalization or death.		4. Physically assaulted, resulting in severe physical injury (e.g., broken bones, deep lacerations, internal injuries); or violent or coercive sexual assault.
	99. Severity not described/provided		99. Severity not described/provided
**[7]**	**Substance Use**	**[8]**	**Suicidal Behavior**
DATE (SOURCE)	☐ NO	DATE (SOURCE)	☐ NO
	1. Uses alcohol.		Demonstrates suicidal behavior = each act in which the adolescent has at least some intention to die, whether or not it would result in death.
	2. Abuses prescribed medication.		
	3. Uses soft drugs (marihuana, hashish, magic mushrooms).		
	4. Uses hard drugs (e.g., GHB, speed, XTC/MDMA, cocaine, LSD/DMT, keta, heroin/opium).		
	5. Uses a drug other than mentioned above (e.g., glue, deodorant, whippets, detergent).		
	99. Substance is not described.		
**[9]**	**Health Neglect**		
PRESENT	☐ NO		
☐ YES	1. Mild problems in one or two domains; hygiene, sleep, diet, or exercise are somewhat below social standards. No implications.		
☐ YES	2. Many problems in self-care (potentially) resulting in moderately negative consequences (e.g., social stigma).		
☐ YES	3. Self-neglecting behavior (potentially) resulting in serious consequences (e.g., not following medical advice, not taking necessary medication, unsafe sexual behavior).		
☐ YES	4. Demonstrates potentially life-threatening behavior (e.g., hunger strikes, not seeking emergency medical treatment).		
☐ YES	99. Severity not described/provided		

START:AV = Short-Term Assessment of Risk and Treatability: Adolescent Version; SOS-AVI = START:AV Outcome Scale: Adolescent Version—Institutional.
