# Supplementary material for: Prospective Field Validation of the START:AV in a Dutch Secure Youth Care Sample
Source: Assessment. 2021 Dec 15;30(3):633–50. doi: 10.1177/10731911211063228 (PMC9999285; doi:10.1177/10731911211063228)
Supplement: sj-docx-1-asm-10.1177_10731911211063228 – Supplemental material for Prospective Field Validation of the START:AV in a Dutch Secure Youth Care Sample [file sj-docx-1-asm-10.1177_10731911211063228.docx]

# Prospective Field Validation of the START:AV in a Dutch Secure Youth Care Sample:

# Supplemental Material

The supplemental material in this document will be made public on Open Science Framework upon acceptance for publication (<https://osf.io/7e2hp>).

[S1. Preregistration 2](#_Toc70591607)

[Table S2 10](#_Toc70591614)

[Table S3 11](#_Toc70591617)

[S4. Additional Coding Instruction for the SOS-AVI 13](#_Toc70591618)

[Table S5 16](#_Toc70591620)

[Table S6 17](#_Toc70591621)

[Table S7 19](#_Toc70591622)

[Table S8 21](#_Toc70591623)

# S1. Preregistration

**Study Information**

**Title**

Predictive Validity of the START:AV for institutional incidents within a Dutch residential youth care sample

**Authors**

(blinded for peer review)

**Hypotheses**

RQ1*: What is the short-term predictive validity of the START:AV for the occurrence of physical aggression, institutional violation, non-violent offenses, substance use, unauthorized absences, suicidal behavior, self-injury, victimization, and health neglect in a residential youth care sample?

A. The Vulnerability total score significantly predicts the presence of SOS-AVI outcomes in a follow-up period of gression, non-violent offenses, institutional violation, substance use, unauthorized leave, health neglect. However, the Vulnerability is not predictive of suicidal behavior, self-injury and, victimization.

B. The Strength total score significantly predicts the absence of SOS-AVI outcomes in a follow-up period of four months, namely physical aggression, non-violent offenses, institutional violation, substance use, unauthorized leave, victimization, health neglect. However, the Strength total score is not predictive of suicidal behavior and self-injury.

C. The specific risk estimates (SRE) on the START:AV significantly predict the respective SOS-AVI outcomes in a follow-up period of four months. More specifically, ‘SRE for Violence’ is predictive of physical aggression; ‘SRE for Non-Violent Offense’ is predictive of non-violent offenses and institutional violation; ‘SRE for Non-Suicidal Self-Injury’ is predictive of self-injury; ‘SRE for Substance Abuse’ is predictive of substance use; ‘SRE for Health neglect’ is predictive of health neglect. However, we hypothesize that there is no significant predictive relationship between the ‘SRE for Suicide’ and suicidal behavior, nor the ‘SRE for Unauthorized Absence’ and unauthorized leave, and the ‘SRE for Victimization’ and victimization.

D. Lifetime history of an adverse outcome is predictive of the corresponding SOS-AVI outcome in a follow-up period of four months. For example, lifetime history of violence rated on the START:AV is predictive of physical aggression rated on the SOS-AVI.

*Hypotheses are based on prior research on the predictive validity of the START:AV (Bhanwer et al., 2016) and research on the predictive validity of the adult START (meta-analysis by O’Shea & Dickens, 2014).

RQ2*: Have the total scores, the prior history and specific risk estimates of the START:AV incremental validity over each other?

A. The specific risk estimate of an adverse outcome has incremental predictive validity over the Vulnerability Total score.

B. The Strength total score has no incremental predictive validity over the Vulnerability Total score.

C. The Vulnerability and Strength total scores have incremental predictive validity over ratings of lifetime history.

*Hypotheses are based on prior research on the predictive validity of the START:AV (Bhanwer et al., 2016) and research on the predictive validity of the adult START (meta-analysis by O’Shea & Dickens, 2014).

RQ3: Is there a gender difference in the predictive accuracy of the START:AV?

A. There are no significant differences in the predictive validity of the START:AV for boys and girls.

**Design Plan**

**Study type**

Observational Study - Data is collected from study subjects that are not randomly assigned to a treatment. This includes surveys, “natural experiments,” and regression discontinuity designs.

**Blinding**

- Personnel who interact directly with the study subjects (either human or non-human subjects) will not be aware of the assigned treatments. (Commonly known as “double blind”)

**Is there any additional blinding in this study?**

The research personnel coding the SOS-AVI from the daily progress notes are blind to the START:AV total scores and specific risk estimates. [double-blind].

**Study design**

This study has a longitudinal prospective design in which adolescents are followed up via digital progress notes for four months after the START:AV completion date.

**Randomization**

Not applicable

**Sampling Plan**

**Existing Data**

Registration prior to analysis of the data

**Explanation of existing data**

The independent variables will be calculated from risk assessment forms (i.e., START:AV forms). These forms have been collected by the first author prior to the preregistration and all data documented on the forms has already been entered in an SPSS file. No data-analysis has occurred prior to preregistration.

**Data collection procedures**

The study is conducted in one of the 14 Dutch residential youth care facilities for youth who are admitted under Dutch civil law with a protection order. Mandated residential youth care is the most restrictive form of residential treatment for adolescents who suffer from severe behavioral and mental health issues. A high level of supervision is required to guarantee their safety (e.g., suicidal behavior or sexual exploitation) and/or the safety of their environment (e.g., violence towards others). At the time of the study, the facility had a capacity of 98 beds: three high-secure (33) and six medium-secure units (65). In 2017, 141 adolescents were admitted for an average duration of 7.9 months. Their average age was 15.6 years old and 43% were girls (Baanders, 2017).

Since 2016, risk assessment with the Short-Term Assessment of Risk and Treatability: Adolescent Version (START:AV; Viljoen, Nicholls, Cruise, Desmarais, & Webster, 2014) is part of standard practice in the facility. The first risk assessment is conducted around 4 weeks after admission on a treatment unit, and 10 weeks on an observation unit. The assessment is repeated every four months. On average, each adolescent will have two risk assessments at time of discharge. With the guidance of the START:AV user guide, the treatment team provides information on the START:AV items, both on the strengths and vulnerabilities of the adolescent. Based on this information and file review, the treatment coordinator rates the items as low, moderate, or high on both the Strength and Vulnerability side (see Appendix A). In the next step, the treatment coordinator codes the history and the risk estimate of the eight adverse outcomes. Treatment coordinators are trained in rating and using the START:AV for treatment purposes.

The START:AV is the subject of this study, rather than the adolescent. The START:AVs which are completed for treatment purposes are gathered by the first author. She oversees the START:AV practice within the facility and has access to all completed START:AVs. In order to assess the predictive validity of the START:AV, we will collect information on institutional incidents in the four months after completion of the START:AV, through file review. Daily progress notes as well as treatment evaluations recorded in the electronic patient file will be accessed and coded on the outcome scale (see measured variables), by a research assistant blind to the START:AV scores.

**Sample size**

N = 160

**Sample size rationale**

Preliminary research on the predictive validity of the START:AV (Viljoen, Beneteau, Gulbransen, et al., 2012) found significant AUC scores ranging from .69 - .82; to detect these AUC values with a power of .80 and an α of .05, a minimum sample size of N = 70 is required (MedCalc version 16.2.0, 2016). Using the minimum in the range of significant effect sizes found in the study of Viljoen and colleagues (r =|.23 - .51|; 2012), with a power of .80 at a two-tailed α of .05; a sample size of N = 143 is required (Faul et al., 2009). When not obstructed by practical issues, we would continue until N = 284 reached because for the hierarchical logistic regression analysis of some adverse outcome a larger sample would be recommended (N between 132 and 284).

**Stopping rule**

Taking into account practical barriers (e.g., time) to data gathering, we will be able to reach N = 160. Then we will stop data gathering. When not obstructed by practical issues, we would continue until N = 284 reached because for the hierarchical logistic regression analysis of some adverse outcome a larger sample would be recommended (N between 132 and 284).

**Variables**

**Manipulated variables**

Not applicable

**Measured variables**

Predictors/independent variables:

1. Strength total score (min 0, max. 48);

2. Vulnerability total score (min 0, max. 48);

3. Lifetime history of adverse outcome (8x; yes/no);

4. Specific risk estimate of adverse outcome (8x; low – moderate – high);

The predictors are collected through the START:AV rating form (Appendix A) in which eight standard adverse outcomes are coded: violence, non-violent offenses, substance abuse, unauthorized absences, suicide, non-suicidal self-injury, victimization, and health neglect.

Outcome variables:

1. Physical aggression

2. Institutional violation

3. Non-violent offenses

4. Substance use

5. Unauthorized leave

6. Self-injury

7. Victimization

8. Suicidal behavior

9. Health neglect

The outcome variables are measured with the START:AV Outcome Scale: Adolescent Version – Institutional (SOS-AVI), an adjusted version of the START Outcome Scale (SOS; Nicholls et al., 2007). The original SOS is based on the Overt Aggression Scale (OAS; Yudofsky, Silver, Jackson, Endicott, & Williams, 1986) which measures four types of aggressive behavior in adults and children: verbal aggression, physical aggression against objects, physical aggression against self, and physical aggression against others. Each outcome has four severity levels on which an incident is rated (1 = least severe, 4 = most severe). The SOS authors supplemented the OAS with the other START adverse outcomes and included an additional outcome sexual violence. Singh and colleagues (2014) slightly modified the SOS for use with the START adolescent version, they added ‘general institutional infraction’. In summary, the SOS for evaluating the START:AV included 12 outcomes, each with four severity levels and one ‘severity not described’ category. Singh et al. (2014) found an interrater reliability of k = .97 (i.e., almost perfect agreement) of the outcome scale, assessed on six cases.

For the present study, the SOS by Singh et al. (2014) has been modified to better fit the START:AV, the adolescent population, and the facility’s rules.

First, in order to be more in line with the adverse outcomes addressed in the START:AV, the SOS categories that were not assessed with the START:AV (e.g., verbal aggression, sexual violence) were omitted in the SOS-AVI, except for institutional infractions. Similar to the SOS, the remaining outcomes have four severity levels and one ‘severity not described’ option.

Second, the outcome ‘non-violent offenses’ was added to the scale. It includes general transgressions and criminal behaviors (e.g., fare dodging, theft, possession of drugs) and it is distinct from institutional violation. Institution violation is rated when an adolescent transgresses specific regulations particular to the facility (e.g., dress code violation, consensual sexual behaviors, smoking in room).

Third, the illustrations provided for each of the severity levels per outcome were fine-tuned to better reflect the outcome descriptions in the START:AV user guide (Viljoen et al., 2014). For example, the former versions of the SOS did not cover absences from school or therapy, although these are types of ‘Unauthorized Leave’ described in the START:AV user guide. In addition, concerning physical aggression, we included the potential, rather than the actual, consequences of the youth’s aggressive incident to rate the severity. Our rational was that a stabbing which was prevented by someone taking the knife should receive the same rating as a stabbing that could not be prevented and caused serious harm.

Fourth, it was decided to rate ‘suicidal behavior’ dichotomously (present/absent) and omit the severity levels as they were difficult to differentiate between (e.g., potentially serious attempt in secluded environment vs. serious attempt with low likelihood of discovery). Moreover, the levels included multiple dimension to rate severity such as lethality and potential for rescue which makes it difficult to rate an incident of which its dimensions can be rated on different severity levels.

Similarly, ‘health neglect’ had multiple dimensions (e.g., domain in which there was neglect, consequences, responsiveness to directions). Therefore, the severity levels were simplified by only focusing on the (potential) consequences. Moreover, each severity level was modified to a dichotomous rating (present/absent), rather than reporting incidents because the neglect typically shows over a period of time.

Last, the SOS-AVI focusses on substance use rather than substance abuse. During trial assessments, it was found difficult to distinguish sufficiently between the severity levels of the SOS’s ‘Substance Abuse’ and its formulations (e.g., ‘occasional’, ‘frequent’, ‘regular’) were deemed incompatible with the approach of reporting separate instances (dates) of substance use/abuse. Therefore, we chose to focus on substance use and distinguished between types of substances. Each incident of substance use is reported in the SOS-AVI. Lastly, the SOS-AVI is formulated in Dutch as this study was conducted in a Dutch sample.

Thus, the SOS-AVI (Appendix B) measures nine outcomes; eight have 4 severity levels and one is rated dichotomously. For seven outcomes (excluding suicidal behavior and health neglect), individual incidents are coded with their respective dates. In the pilot phase (n=6), the average measure interrater reliability (ICC2) of the scale was excellent for all outcomes (ranging from .80 to 1.00), except for institutional violation (ICC2 = .627) and health neglect (ICC2 = .348). The single measure interrater reliability (ICC1) was excellent for physical aggression (ICC1 = 1.00), non-violent offenses (ICC1 = .93), substance use (ICC1 = .95), and self-injury (ICC1 = .80); good for unauthorized leave (ICC1 = .67) and victimization (ICC1 = .67), fair for institutional violation (ICC1 = .46) and poor for health neglect (ICC1 = .21). No suicidal behaviors were rated during this pilot test. Following this test of interrater reliability, the rating criteria for institutional violation and health neglect were discussed and further clarified in the coding manual. Interrater reliability will be reassessed during data gathering.

In sum, for each dependent variable (i.e., SOS-AVI outcome) four predictors are collected from the START:AV forms.

Moderating variable: gender (male / female).

**Indices**

The Strength total score will be calculated as the sum of 24 Strength items (rated as 0, 1, or 2) in the START:AV rating form.

The Vulnerability total score will be calculated as the sum of 24 Vulnerability items (rated as 0, 1, or 2) in the START:AV rating form.

The START:AV variables ‘recent history’ and ‘prior history’ will be combined into one ‘lifetime history’ of adverse outcome (Yes/No). When at least one of recent/prior history is present, the ‘history’ variable will be coded as present.

SOS-AVI outcomes will be calculated as the sum of all incidents on five (4+1) severity scales and will be converted into present/absent.

**Analysis Plan**

**Statistical models**

**1. Descriptive statistics**

〉- Mean (standard deviation) and range of the START:AV items

〉- Endorsement frequencies of the START:AV items ratings

〉- Endorsement frequencies of history and specific risk estimates of the adverse outcomes

〉- Percentage of youth with 1, 2, 3 or more high specific risk estimates

〉- Frequency of missing values per item

〉- Mean (standard deviation) and range of the number of incidents per SOS-AVI outcome

〉- Distribution of the incidents across severity levels per SOS-AVI outcome

〉- Prevalence rates of SOS-AVI outcomes in the sample

**2. Descriptive: correlations between**

〉 - strength and vulnerability item ratings (Kendall Tau correlations);

〉 - strength and vulnerability total scores (Pearson correlation coefficient);

〉 - item ratings and specific risk estimates (Kendall Tau correlations);

〉 - total scores and specific risk estimates (Kendall Tau correlations);

〉 - Specific risk estimates (Kendall Tau correlations)

〉 - SOS outcomes (present/absent) and the strength total score (Biserial correlation);

〉 - SOS outcomes (present/absent) and the vulnerability total score (Biserial correlation);

〉 - SOS outcomes (present/absent) and the specific risk estimates (Chi-square test).

**3. Gender differences:**

〉 - Chi-square test to assess gender differences in item ratings

〉 - T-test to assess gender differences in total scores

〉 - Chi-square test to assess gender differences in specific risk estimates

Prior to testing predictive validity, we will test whether gender is a moderating variable for the predictive validity of total scores, history or specific risk estimates. This will be examined in a stepwise multiple regression. When a significant moderator effect is found, the analyses will be conducted for male and female adolescents separately. When there is no moderating effect of gender, the samples will be collapsed (Viljoen et al., 2012).

**4. Predictive validity:**

Although predictive validity of risk assessment instruments is most commonly measured with a ROC curve analysis, there are various approaches and various performance indicators available that measure different aspects of predictive validity. In general, there are two components to predictive validity: discrimination and calibration (Singh, 2013). Discrimination refers to how well an instrument is able to separate those who went on to be violent from those who did not (Cook, 2007), whereas calibration refers to how well the risk predictions (probabilities) of an instrument align with the actual observed incidents (proportion). To evaluate the prognostic value of an instrument, both should be assessed. Measures of discrimination include sensitivity and specificity, odds ratio, and ROC curve. Indicators of calibration include positive and negative predictive power. In this study, the following analyses of predictive accuracies will be conducted:

Measures of discrimination

A. Sensitivity and specificity of each START:AV adverse outcome

a. Sensitivity: 2 (rule-in risk bin, see below) x2 (presence/absence outcome) contingency table

b. Specificity: 2 (rule-out risk bin, see below) x2 (presence/absence outcome) contingency table

c. Likelihood ratios (LR+ and LR -)

B. ROC Curve analysis: Predictive accuracy of occurrence outcome

d. Vulnerability total scores -> presence/absence of outcome

e. Strength total scores -> presence/absence of outcome

f. History of outcome -> presence/absence of outcome

g. Specific risk estimate -> presence/absence of outcome

Measures of calibration

C. Positive and negative predictive value

h. Positive predictive value: TP/(TP+FP)

i. Negative predictive value: TN/(TN + FN)

**5. Incremental predictive validity**

A. Hierarchical Binary logistic regression for each Adverse Outcome with

Block 1: Vulnerability total score

Block 2: Vulnerability total score + SRE for Adverse Outcome X

B. Hierarchical Binary logistic regression for each Adverse Outcome with

Block 1: Strength total score

Block 2: Strength total score + SRE for Adverse Outcome X

C. Hierarchical Binary logistic regression for each Adverse Outcome with

Block 1: Vulnerability total score

Block 2: Vulnerability total score + Strength total score

Block 3: Vulnerability total score + Strength total score + SRE for Adverse Outcome X

D. Hierarchical Binary logistic regression for each Adverse Outcome with

Block 1: History of Adverse Outcome X

Block 2: History of Adverse Outcome X + Vulnerability total score + Strength total score

**Transformations**

AUC scores for the Strength total scores will be reverted to predict the absence of outcomes.

START:AV specific risk estimates will be recoded from Low (0), Moderate (1), High (2) to ‘presence (1) / absence (0)’ depending on the risk binning strategy:

Calculation of sensitivity & specificity, odds ratios, and positive and negative predictive power requires ‘risk binning’ to dichotomize the specific risk estimates (Singh et al., 2011). The first risk bin strategy is grouping individuals with low and moderate specific risk estimates and compare them with those with a high specific risk estimate (‘rule-in decision’). This strategy is relevant when high sensitivity is desired, such as for screening purposes. The second strategy is grouping individuals with moderate and high risk and compare them to the individuals with a low specific risk estimate (‘rule-out decision’). This strategy is relevant when high specificity is required, for example to identify specific cases.

**Inference criteria**

1. Correlations:

A. Pearson r and biserial correlations: * p < .05. ** p < .01, two-tailed; |.10|= small correlation; |.30|= moderate correlation; |.50 to 1.0|= large correlation (Cohen, 1988).

B. Kendall Tau correlations: * p < .05. ** p < .01, two-tailed

2. ROC curve analysis:

A. AUC value: * p < .05. ** p < .01, two-tailed; an AUC value can be interpreted based on benchmarks that indicate a small, moderate or large effect. However, these are not supposed to be used in the context of predictive models in risk assessment (Singh, 2013). Therefore, we will not use the benchmarks to interpret the AUCs. We will compare the AUC values with values found in previous studies.

3. Multiple testing correction: For each research question, we will correct for multiple testing using the Bonferroni correction. For the first research question on predictive accuracy, we count three tests per separate outcome, therefore, we will use α of 0.016 (0.05/3). For the second research question on incremental predictive accuracy, an α of 0.013 is required since we will conduct four tests per outcome. For all hypotheses, we will report both the corrected and non-corrected results.

**Data exclusion**

From the total dataset with START:AVs completed from February 1, 2016 until December 31, 2018 (N = 477), the following START:AVs will be excluded:

1. Cases completed with the purpose of ‘double coding’, as these START:AVs were not used for treatment purposes.

2. Cases completed by trainees.

3. Cases completed by treatment coordinators who were not trained in the START:AV.

4. Cases in which the youth was discharged within 17 weeks after the START:AV completion date as we wish to assess the SOS-AVI outcomes over a four month period (or 17 weeks).

5. Cases completed on non-mandated residential youth care units as we focus on mandated youth care.

6. Cases of which the adolescent primarily (i.e., more than 10 weeks) resided on a high secure unit during the 17 week period.

**Missing data**

Missing data of independent variables (START:AVS)

- Missing data can occur for Strength and Vulnerability item ratings (low moderate high), specific risk estimate ratings (low moderate high) and History ratings (presence/absence).

- Missing data occurs when a START:AV assessor forgets to mark a rating or when the assessor deems there is too little information to rate the item.

When there is missing data in a case, we first go back to the START:AV rating form and manually check the missing information to assess whether the information is truly missing or the rater forgot to mark the bullet. Especially with historical information, it occurs that the rater writes down historical information on an adverse outcome but forgets to mark ‘History’ as present. When this is the case, this missing information will be added to the dataset.

Missing information that could not be retrieved, will be handled according to the multiple imputation approach (Rubin, 1977). This will be done for cases with 4 or less (20%) missing Strength and/or Vulnerability ratings. Cases with five (i.e., approximately 20%) or more missing Strength ratings and/or five or more missing Vulnerability ratings will be excluded from analysis. We copied this rule from prior research with the START:AV (Viljoen et al., 2012). This cut-off percentage is typically used in research (Mazza, Enders, & Ruehlman, 2015).

Missing data of dependent variables (SOS-AVI outcomes): Cases with missing specific risk estimates will be excluded from analysis of that particular adverse outcome.

**Exploratory analysis**

Recording dates and severity of incidents on the SOS-AVI leaves the opportunity to further analyze the outcome data according to severity or proximity in time (e.g. survival analysis). However, this is beyond the scope of this study.

**Other**

**References**

Baanders, A. N. (2017). *JeugdzorgPlus-behandeling bij de OG Heldringstichting* [Treatment in secure youth care at the OG Heldring Institution]. OG Heldringstichting: unpublished.

Bhanwer, A., Shaffer, C., & Viljoen, J. L. (2016). Short-term Assessment of Risk and Treatability: Annotated Bibliography. Burnaby: British Columbia University.

Faul, F., Erdfelder, E., Buchner, A. & Lang, A.G. (2009) Statistical Power Analyses Using G*Power 3.1: Tests for Correlation and Regression Analyses. *Behavior Research Methods*, *41*, 1149-1160. doi: http://dx.doi.org/10.3758/BRM.41.4.1149

Mazza, G.L., Enders, C. K., & Ruehlman, L. S. (2015). Addressing item-level missing data: a comparison of proration and full information maximum likelihood estimation. *Multivariate Behavioural Research, 50*(5), 504-519. doi: 10.1080/00273171.2015.1068157

Nicholls, T., Gagnon, N., Crocker, A., Brink, J., Desmarais, S., & Webster, C. (2007). *START Outcomes Scale (SOS)*. Vancouver, Canada: BC Mental Health & Addiction Services.

O’Shea, L. E., & Dickens, G. L. (2014) Short-Term Assessment of Risk and Treatability (START): systematic review and meta-analysis. *Psychological Assessment, 26*, 990-1002.

Singh, J. P., Desmarais, S. L., Hurducas, C., Arbach-Lucioni, K., Condemarin, C., Dean, K., … Otto, R. K. (2014). International perspectives on the practical application of violence risk assessment: A global survey of 44 countries. *International Journal of Forensic Mental Health, 13*(3), 193 – 206. doi: 10.1080/14999013.2014.922141

Singh, J. P. (2013). Predictive validity performance indicators in violence risk assessment: a methodological primer. Behavioral Sciences and the Law, 31, 8-22. doi: 10.1002/bsl.2052

Singh, J. P., Desmarais, S. L., Sellers, B. G., Hylton, T., Tirotti, M., & Van Dorn, R. A. (2014). From risk assessment to risk management: Matching interventions to adolescent offenders’ strengths and vulnerabilities. *Children and Youth Services Review, 47,* 1 – 9. doi: 10.1016/j.childyouth.2013.09.015

Viljoen, J. L., Nicholls, T. L., Cruise, K. R., Desmarais, S. L., & Webster, C. D. with contributions by Douglas-Beneteau, J. (2014). *Short-Term Assessment of Risk and Treatability: Adolescent Version (START:AV) – User Guide*. Burnaby, British Columbia: Mental Health, Law, and Policy Institute.

Viljoen, J. L., Beneteau, J. L., Gulbransen, E., Brodersen, E., Desmarais, S. L., Nicholls, T. L., & Cruise, K. R. (2012). Assessment of multiple risk outcomes, strengths, and change with the START:AV: A short-term prospective study with adolescent offenders. *International Journal of Forensic Mental Health, 11,* 165-180. doi: 10.1080/14999013.2012.737407

Yudofsky, S. C., Silver, J. M., Jackson, W., Endicott, J., & Williams, D. (1986). The Overt Aggression Scale for the objective rating of verbal and physical aggression. *The American Journal of Psychiatry, 143,* 35-39.

Table S2**.**

*Adjustments From the Preregistered Plan*

| Introduction |
| --- |
| 1. Due to page constraints, detailed hypotheses were not included in the paper and only the overall research questions were addressed in the section ‘Present Study’. 2. The research question on gender differences in the predictive validity of the START:AV was not addressed in this study to keep the number of analyses manageable. Furthermore, the sample size was too small to split the sample into boys and girls. As a result gender was not assessed as moderating variable. |
| Method |
| 1. In the preregistration, we stated that the START:AV consists of 24 items. However, given that two items have sub-items (i.e., item 13a and 13b, and item 14a and 14b); the total number of START:AV items is 26. This was clarified in the paper. 2. In hindsight, the preregistration included too many analyses, therefore, secondary analyses were not conducted. Excluded analyses are:    1. Percentage of youth with 1, 2, 3 or more high specific risk estimates    2. Correlations between item ratings and specific risk estimates    3. Correlations between specific risk estimates    4. Sensitivity and specificity    5. Likelihood ratios    6. Positive and negative predictive value 3. Predictive validity of lifetime history could not be assessed with ROC curve analysis but was assessed using logistic regression and odds ratios were reported. 4. Bonferroni correction was deemed not necessary for the current analyses, because the multiple analyses each apply to different dependent variables. Additionally, the Bonferroni correction is generally not applied in (hierarchical) regression analyses. |

Table S3**.**

*Modifications to the START Outcome Scale (SOS) adolescent version resulting in the START:AV Outcome Scale: Institutional (SOS-AVI)*

|  | Modifications |
| --- | --- |
|  | Adverse outcomes for which the START:AV was not designed to assess, were excluded as a SOS category. That is, verbal aggression, suicide ideation and planning, and sexual violence were omitted in the SOS-AVI, except for institutional infractions. Violence against property was omitted as a separate category and is captured in the ‘non-violent offenses’ category. |
|  | The START:AV outcome ‘non-violent offenses’ was added. It includes general transgressions and criminal behaviors (e.g., fare dodging, theft, possession of drugs, vandalism) and it is distinct from institutional violations. Institution violation is rated when an adolescent transgresses specific regulations particular to the facility (e.g., dress code violation, consensual sexual behaviors, smoking in room), whereas non-violent offenses are transgressions of societal rules and laws. |
|  | Examples per severity level of the outcome categories were fine-tuned to better reflect the outcome descriptions in the START:AV user guide (Viljoen et al., 2014). For example, the former versions of the SOS did not cover absences from school or therapy, although these are types of ‘Unauthorized Leave’ described in the START:AV user guide. In addition, for physical aggression, we included *potential*, rather than actual, consequences of the youth’s aggressive incident to rate the severity. That is, an prevented attempt to stab someone received the same severity rating as a stabbing that was not prevented and caused serious harm. |
|  | For the outcome ‘suicidal behavior’, the levels of severity were omitted and a dichotomous rating (present/absent) was introduced. In practice, it proved difficult to differentiate between the severity levels (e.g., the severity of ‘potentially serious attempt in secluded environment’ versus ‘serious attempt with low likelihood of discovery’). Moreover, the description of a severity level was based on more than one indicator (e.g., lethality risk, potential for rescue) which made it difficult to rate an incident when the incident had indicators that could be coded under different levels. |
|  | Similarly, ‘health neglect’ included multiple indicators (e.g., health domain, consequences of neglect, responsiveness to directions). Therefore, the indicators to differentiate between severity levels were simplified by only addressing the (potential) consequences. Because neglect typically showed over a period of time (rather than incidental), the severity levels were modified to a dichotomous rating (present/absent). |
|  | The adjusted SOS-AVI focusses on substance *use* rather than *abuse*. During trial assessments, it was found difficult to distinguish sufficiently between the severity levels of the SOS’s ‘Substance Abuse’ (e.g., difference between mild and moderate impairments). Moreover, the indicators ‘occasional’, ‘frequent’, and ‘regular’ were incompatible with reporting separate incidents (dates) of substance intake. Therefore, we decided to code each substance use incident and differentiate between types of substances. |

# S4. Additional Coding Instruction for the SOS-AVI

## Clarification Header

Record each incident with the date followed by the source. If the incident is from a daily progress note, then you do not need to list the source. Use (*TBP*) when the source is a treatment plan and (I) for incident form as source. Example: 01.02.2017 (I).

Discharged youth: If the adolescent has been discharged before the end of the SOS period then the SOS will not be completed. A margin of a few days is allowed. Document this by noting the discharge date. If unknown, record the date of last daily progress note.

Group SOS period: Before you start reading the report, first take a look at the adolescent's timeline in MC4. Check which security level the adolescent was at for the longest time during the SOS period. Codes V2.1 and V2.2 mean high secure, codes V2.4, V2.6, V2.7 mean medium secure, codes V2.8 and 3 represent low secure units. Also check to see if the adolescent has been transferred in between.

Transferred youth: If the timeline shows that the adolescent has been transferred to a different level of security (e.g., from medium secure V2.6 to high secure V2.1) during the SOS period, check this box on the SOS form. Mark which security level the youth transferred to and when. In the SOS database (SPSS), this is tracked under the variable 'SOS security'. There you indicate whether the adolescent stayed mainly on a high secure unit (at least 2.5 months), or on a medium secure unit (at least 2.5 months), or low secure unit (at least 2.5 months).

**General Information**

Severity: Each SOS outcome consists of four levels of severity. Examples are provided for each severity level. If the severity is not clear from the report, record the date of the incident (or the check mark) under 'severity unknown'. If multiple severity levels of a SOS outcome are present in one incident, code only the most severe aspect of the incident.

Date: record the date the incident occurred. If this is unknown, record the date the incident was reported. If this is also unknown, record the month. The incident must have occurred during the SOS period.

- Sometimes a daily progress note will have two different dates. Always take the date in the title of the progress note. If there is no date and it cannot be deduced from the progress note, then take the automatically created date (i.e., date of uploading the dialy progress note).
- Record 1 date per incident. If there were similar separate incidents that day, note how often. For example: 27.02.2018 (x2) and note them in the table for the excerpts.
- Tip: When you read about an incident, note it immediately in the SOS scale (including the date). Do not wait until you have read the progress note completely.

Suspicions: Suspicions of harmful outcomes are not coded in the SOS scale. An incident must be confirmed by the adolescent and/or (reliable) witness(es) to the incident (i.e., in the same progress note or in reports documented in the following days). Exceptions may be made for suspicions of substance use under certain conditions. When typical signs of substance abuse are observed and reported by group care workers and the suspicion is followed up with actions (e.g., UC, restrictions), then the suspicion of substance use may be coded as an incident (except if the UC proved negative).

Intention: Code based on behavior you observe, independent of the intentions of the adolescent may have had. Only suicidal behavior should have suicidal intent present.

**Information SOS-AVI Outcomes**

1. Physical aggression: If the report speaks of a "physical conflict" but this is not further specified, code this as 'severity unknown'. Code only if the physical aggression or threat of physical harm is directed toward a specific other.

- Code if adolescent wants to attack physically but is stopped in time;
- Code if the adolescent shows physical aggression back in defense
- DO NOT code if adolescent is only a victim and not a perpetrator.
- DO NOT code if the behaviors was meant to be playful or teasing (e.g., playful kick under the table).

1. Institutional Violation: Code all behavior that is against the rules, regardless of the underlying intent. If the institutional violation can also be coded as another outcome (e.g., non-violent offense), then only code the incident under the **other** outcome. Code failure to follow dress code only when the youth openly refuses to follow the dress code (and does not change clothes after a warning). When it says that adolescents kissed each other and it is not clear if it was on the mouth, you give the benefit of the doubt and do not code this as a sexual act. A kiss on the mouth is prohibited at the institution.
   - Prohibited items (not drugs or weapons): e.g., tabacco, lighter, money, aerosol (deodorant), etc. without permission from group care workers.

1. Non-violent offenses:

- Nuisance: Code only behavior that others complain about and are clearly bothered by, this can be loud music or prolonged loud shouting requiring intervention from staff.
- Vandalism: Deliberate destruction of items belonging to someone else is coded as a non-violent offense. Depending on the severity of the vandalism, it is coded as Level 2 (e.g., puncturing bike tires) or Level 3 (e.g., vandalizing a bus shelter).
- Illegal graffiti: Like vandalism, the deliberate and unauthorized daubing of walls and furniture is coded as a non-violent offense.
- Youth Recruiting: If there is evidence that an adolescent is recruiting other youth at the facility for loverboys/human traffickers (e.g., forcing to connect with them), code this as level 4.

1. Absences: Returning late is only coded when the adolescent is significantly late (e.g., 10 minutes late is not coded). Not following the daily structure or not going to appointments other than treatment appointments are NOT coded.

- Deregistration: Absconding resulting in deregistration from the facility and the on-site school is coded under level 4.
- Absence from school: If an adolescent is absent from school because they are sleeping in, this is an absence without a valid reason and is coded as unauthorized absence (unless permission is given by group care workers). When the adolescent is send back from school to the unit as a preventive measure (e.g. because of rising tension), or when the adolescent makes a substitute school assignment on the unit, this is not coded as an unauthorized absence. When adolescent is expelled from class because of disruptive behavior, this is also not coded; however, leaving the class without permission is an unauthorized absence.

1. Self-harm: Code any behavior in which the adolescent intentionally harms themselves. Consider the severity of the damage. For example, if severe head-banging resulted in unconsciousness, code this at level 4 (instead of 2). When ingesting poison (without suicidal intention), try to find out how harmful the poison was. If unknown, code at 99.

- Difference between scratching and cutting: Superficial scratching is when the scratching results in red welts (level 1). Scratching with moderate damage is when the wound needs care such as disinfection (level 3). Scratching (= cutting) with severe damage is when it needs stitches (level 4). When superficial scratching is reported, but bandages were needed, you code it at level 3 instead of 1. A cut that required stitches counts as a deep cut (level 4).

1. Victimization: Code the adolescent's experienced victimization as a result of an event that occurred during the SOS period, and not victimization as a result of past trauma. If an adolescent is actively excluded by others and they experience some distress as a result, then code this as level 1. If there are self-neglecting behaviors in the context of victimization (e.g., not eating because of being locked in somewhere by others), do not code this at self-neglect.
2. Substance Use: Code any event in which the adolescent used the listed drugs. If multiple substances were used in one situation, code the highest level.
3. Suicidal Behavior: Code any behavior in which the adolescent did something to themselves with the intent to die, including the date it took place.
4. Health Neglect: Code for the entire period (no dates) and if multiple forms are present, code the most severe form. Guideline: If a minimal form of health neglect (e.g. refusing to eat one meal) is reported several times during the SOS period then you can code this. Especially if it is supported by information from, for example, the medical service. However, if it is mentioned once, then this is not sufficient to code as self-neglect.

- Refusing medication: If the adolescent refuses their medication, code this as health neglect only if the refusal of medication could have a major impact on the adolescent (mental) health (e.g., antipsychotics vs. anticonception).

Table S5**.**

*Interrater Reliability of the SOS-AVI Outcomes*

| SOS-AVI outcome | ICC | ICC Interpretation | CI lower bound | CI upper bound | CI Interpretation |
| --- | --- | --- | --- | --- | --- |
| Physical Aggression | 1.00 | Excellent | 1.00 | 1.00 | Excellent |
| Non-Violent Offenses | .97 | Excellent | .94 | .99 | Excellent |
| Institutional Violation | .94 | Excellent | .88 | .97 | Good to Excellent |
| Substance Use | .96 | Excellent | .92 | .98 | Excellent |
| Unauthorized Leave | .91 | Excellent | .82 | .96 | Good to Excellent |
| Suicidal Behavior ^a^ | - | - | - | - | - |
| Self-Injury | .99 | Excellent | .98 | 1.00 | Excellent |
| Victimization | .88 | Good | .76 | .94 | Good to Excellent |
| Health Neglect | .93 | Excellent | .86 | .97 | Good to Excellent |
| Total incidents | .97 | Excellent | .94 | .99 | Excellent |

*Note*. *N* = 30. ICC = two-way random, absolute agreement, single measure intraclass correlation coefficient. CI = confidence interval.

^a^ No incidents relevant to this outcome were coded.

Table S6**.**

*Descriptive Statistics, Frequency of Endorsement, and Correlations of the START:AV Items*

| START:AV item |  | Descriptives | | | | Frequency | | | Correlation |
| --- | --- | --- | --- | --- | --- | --- | --- | --- | --- |
|  |  | N | Mean | Sd | Missing | Low | Moderate | High | (k-Tau) |
| School and Work | Strength | 105 | 0.88 | 0.72 | 1 | 32.4 | 47.6 | 20.0 | -.47*** |
|  | Vulnerability | 102 | 1.13 | 0.58 | 4 | 10.8 | 65.7 | 23.5 |  |
| Recreation | Strength | 105 | 0.81 | 0.59 | 1 | 28.6 | 61.9 | 9.5 | -.29** |
|  | Vulnerability | 103 | 1.14 | 0.51 | 3 | 6.8 | 72.8 | 20.4 |  |
| Substance use | Strength | 104 | 0.74 | 0.76 | 2 | 45.2 | 35.6 | 19.2 | -.52*** |
|  | Vulnerability | 106 | 0.96 | 0.79 | 0 | 33.0 | 37.7 | 29.2 |  |
| Rule Adherence | Strength | 105 | 0.71 | 0.62 | 1 | 37.1 | 54.3 | 8.6 | -.25** |
|  | Vulnerability | 105 | 1.41 | 0.55 | 1 | 2.9 | 53.3 | 43.8 |  |
| Conduct | Strength | 104 | 0.75 | 0.52 | 2 | 28.8 | 67.3 | 3.8 | -.22* |
|  | Vulnerability | 105 | 1.46 | 0.57 | 1 | 3.8 | 46.7 | 49.5 |  |
| Self-Care | Strength | 105 | 1.01 | 0.63 | 1 | 19.0 | 61.0 | 20.0 | -.36*** |
|  | Vulnerability | 105 | 1.06 | 0.60 | 1 | 15.2 | 63.8 | 21.0 |  |
| Coping | Strength | 103 | 0.39 | 0.51 | 3 | 62.1 | 36.9 | 1.0 | -.37*** |
|  | Vulnerability | 105 | 1.71 | 0.53 | 1 | 3.8 | 21.0 | 75.2 |  |
| Impulse Control | Strength | 101 | 0.60 | 0.65 | 5 | 48.5 | 42.6 | 8.9 | -.40*** |
|  | Vulnerability | 106 | 1.27 | 0.63 | 0 | 9.4 | 53.8 | 36.8 |  |
| Mental state | Strength | 104 | 0.71 | 0.66 | 2 | 40.4 | 48.1 | 11.5 | -.33*** |
|  | Vulnerability | 106 | 1.22 | 0.62 | 0 | 10.4 | 57.5 | 32.1 |  |
| Emotional state | Strength | 103 | 0.50 | 0.54 | 3 | 51.5 | 46.6 | 1.9 | -.21* |
|  | Vulnerability | 104 | 1.61 | 0.57 | 2 | 3.8 | 31.7 | 64.4 |  |
| Attitudes | Strength | 99 | 0.73 | 0.60 | 7 | 35.4 | 56.6 | 8.1 | -.28** |
|  | Vulnerability | 104 | 1.42 | 0.65 | 2 | 8.7 | 40.4 | 51.0 |  |
| Social Skills | Strength | 106 | 0.93 | 0.61 | 0 | 21.7 | 63.2 | 15.1 | -.41*** |
|  | Vulnerability | 104 | 1.14 | 0.58 | 2 | 10.6 | 64.4 | 25.0 |  |
| Relationships Adults | Strength | 102 | 0.88 | 0.59 | 4 | 23.5 | 64.7 | 11.8 | -.40*** |
|  | Vulnerability | 104 | 1.38 | 0.64 | 2 | 8.7 | 45.2 | 46.2 |  |
| Relationships Peers | Strength | 101 | 0.63 | 0.63 | 5 | 44.6 | 47.5 | 7.9 | -.21* |
|  | Vulnerability | 104 | 1.27 | 0.60 | 2 | 7.7 | 57.7 | 34.6 |  |
| Social Support Adults | Strength | 101 | 0.69 | 0.69 | 5 | 43.6 | 43.6 | 12.9 | -.42*** |
|  | Vulnerability | 99 | 1.14 | 0.64 | 7 | 14.1 | 57.6 | 28.3 |  |
| Social Support Peers | Strength | 99 | 0.33 | 0.55 | 7 | 70.7 | 25.3 | 4.0 | -.30** |
|  | Vulnerability | 100 | 1.42 | 0.54 | 6 | 2.0 | 54.0 | 44.0 |  |
| Parenting | Strength | 103 | 0.71 | 0.68 | 3 | 41.7 | 45.6 | 12.6 | -.49*** |
|  | Vulnerability | 102 | 1.41 | 0.64 | 4 | 7.8 | 43.1 | 49.0 |  |
| Parental Functioning | Strength | 99 | 0.71 | 0.61 | 7 | 37.4 | 54.5 | 8.1 | -.34*** |
|  | Vulnerability | 97 | 1.26 | 0.65 | 9 | 11.3 | 51.5 | 37.1 |  |
| Peers | Strength | 95 | 0.27 | 0.52 | 11 | 75.8 | 21.1 | 3.2 | -.16 |
|  | Vulnerability | 96 | 1.34 | 0.61 | 10 | 7.3 | 51.0 | 41.7 |  |
| Material Resources | Strength | 104 | 1.06 | 0.41 | 2 | 5.8 | 82.7 | 11.5 | -.40*** |
|  | Vulnerability | 102 | 0.76 | 0.51 | 4 | 27.5 | 68.6 | 3.9 |  |
| Community | Strength | 99 | 1.01 | 0.30 | 7 | 4.0 | 90.9 | 5.1 | -.23* |
|  | Vulnerability | 99 | 0.98 | 0.29 | 7 | 5.1 | 91.9 | 3.0 |  |
| External Triggers | Strength | 96 | 0.31 | 0.51 | 10 | 70.8 | 27.1 | 2.1 | .32** |
|  | Vulnerability | 99 | 0.72 | 0.77 | 7 | 47.5 | 33.3 | 19.2 |  |
| Insight | Strength | 104 | 0.76 | 0.65 | 2 | 35.6 | 52.9 | 11.5 | -.35*** |
|  | Vulnerability | 102 | 1.31 | 0.61 | 4 | 7.8 | 52.9 | 39.2 |  |
| Plans | Strength | 98 | 0.81 | 0.70 | 8 | 35.7 | 48.0 | 16.3 | -.48*** |
|  | Vulnerability | 99 | 1.05 | 0.61 | 7 | 16.2 | 62.6 | 21.2 |  |
| Medication Adherence ^a^ | Strength | 33 | 1.06 | 0.70 | 7 | 21.2 | 51.5 | 27.3 | -.45** |
|  | Vulnerability | 35 | 0.57 | 0.61 | 5 | 48.6 | 45.7 | 5.7 |  |
| Treatability | Strength | 100 | 0.79 | 0.70 | 6 | 37.0 | 47.0 | 16.0 | -.40*** |
|  | Vulnerability | 100 | 1.17 | 0.60 | 6 | 11.0 | 61.0 | 28.0 |  |

^a^ with *n* = 66 completed as ‘not applicable’

* *p* < .05, ** *p* < .01, *** *p* < .001

Table S7**.**

*Prevalence of Adverse Outcomes and Unique Incidents During Follow-Up, Including Severity Levels*

|  | Presence | |  | Unique Incidents | | | |
| --- | --- | --- | --- | --- | --- | --- | --- |
|  | *n* | % |  | *M* | *SD* | Range | Total |
| Any Incidents | 105 | 99.1 |  | 28.92 | 19.14 | 0-91 | 3065 |
| Physical aggression | 78 | 73.6 |  | 3.85 | 4.89 | 0-26 | 408 |
| Severity Level 1 | 62 | 58.5 |  | 2.07 | 3.53 | 0-21 | 219 |
| Severity Level 2 | 50 | 47.2 |  | 0.93 | 1.31 | 0-7 | 99 |
| Severity Level 3 | 21 | 19.8 |  | 0.29 | 0.66 | 0-3 | 31 |
| Severity Level 4 | 16 | 15.1 |  | 0.27 | 0.85 | 0-6 | 29 |
| Unknown severity | 23 | 21.7 |  | 0.28 | 0.58 | 0-2 | 30 |
| Non-violent offenses | 88 | 83.0 |  | 5.28 | 5.29 | 0-24 | 560 |
| Severity Level 1 | 75 | 70.8 |  | 2.84 | 3.16 | 0-16 | 301 |
| Severity Level 2 | 74 | 69.8 |  | 1.99 | 2.32 | 0-11 | 211 |
| Severity Level 3 | 1 | 0.9 |  | 0.01 | 0.10 | 0-1 | 1 |
| Severity Level 4 | 22 | 20.8 |  | 0.39 | 0.98 | 0-7 | 41 |
| Unknown severity | 5 | 4.7 |  | 0.06 | 0.27 | 0-2 | 6 |
| Institutional violation | 98 | 92.5 |  | 8.47 | 7.82 | 0-43 | 898 |
| Severity Level 1 | 67 | 63.2 |  | 1.72 | 2.28 | 0-15 | 182 |
| Severity Level 2 | 76 | 71.7 |  | 2.67 | 3.25 | 0-24 | 283 |
| Severity Level 3 | 79 | 74.5 |  | 3.71 | 4.68 | 0-24 | 393 |
| Severity Level 4 | 23 | 21.7 |  | 0.32 | 0.74 | 0-4 | 34 |
| Severity unknown | 5 | 4.7 |  | 0.06 | 0.27 | 0-2 | 6 |
| Substance use | 59 | 55.7 |  | 2.37 | 3.33 | 0-15 | 251 |
| Alcohol | 13 | 12.3 |  | 0.18 | 0.53 | 0-3 | 19 |
| Medication | 3 | 2.8 |  | 0.04 | 0.24 | 0-2 | 4 |
| Soft drugs | 54 | 50.9 |  | 1.85 | 2.71 | 0-12 | 196 |
| Hard drugs | 14 | 13.2 |  | 0.17 | 0.49 | 0-3 | 18 |
| Other | 6 | 5.7 |  | 0.12 | 0.64 | 0-5 | 13 |
| Substance Unknown | 9 | 8.5 |  | 0.13 | 0.50 | 0-3 | 14 |
| Unauthorized leave | 91 | 85.8 |  | 4.14 | 4.58 | 0-28 | 439 |
| Severity Level 1 | 79 | 74.5 |  | 2.93 | 3.41 | 0-17 | 311 |
| Severity Level 2 | 21 | 19.8 |  | 0.48 | 1.39 | 0-10 | 51 |
| Severity Level 3 | 24 | 22.6 |  | 0.25 | 0.50 | 0-2 | 27 |
| Severity Level 4 | 29 | 27.4 |  | 0.45 | 0.87 | 0-4 | 48 |
| Unknown severity | 2 | 1.9 |  | 0.02 | 0.14 | 0-1 | 2 |
| Suicidal behavior | 4 | 3.8 |  | 0.05 | 0.25 | 0-2 | 5 |
| Self-injury | 44 | 41.5 |  | 2.05 | 4.44 | 0-30 | 217 |
| Severity Level 1 | 25 | 23.6 |  | 0.48 | 1.15 | 0-7 | 51 |
| Severity Level 2 | 21 | 19.8 |  | 0.40 | 1.02 | 0-6 | 42 |
| Severity Level 3 | 22 | 20.8 |  | 0.69 | 1.78 | 0-9 | 73 |
| Severity Level 4 | 11 | 10.4 |  | 0.08 | 0.31 | 0-17 | 42 |
| Unknown severity | 8 | 7.5 |  | 0.40 | 1.89 | 0-2 | 9 |
| Victimization | 67 | 63.2 |  | 2.26 | 3.16 | 0-15 | 240 |
| Severity Level 1 | 48 | 45.3 |  | 1.40 | 2.27 | 0-11 | 148 |
| Severity Level 2 | 40 | 37.7 |  | 0.58 | 0.98 | 0-6 | 62 |
| Severity Level 3 | 17 | 16.0 |  | 0.24 | 0.63 | 0-4 | 25 |
| Severity Level 4 | 3 | 2.8 |  | 0.03 | 0.17 | 0-1 | 3 |
| Unknown severity | 2 | 1.9 |  | 0.02 | 0.14 | 0-1 | 2 |
| Health neglect^1^ | 47 | 44.3 |  |  |  |  | 47 |
| Severity Level 1 | 19 | 17.9 |  |  |  |  | 19 |
| Severity Level 2 | 12 | 11.3 |  |  |  |  | 12 |
| Severity Level 3 | 16 | 15.1 |  |  |  |  | 16 |
| Severity Level 4 | 0 | 0 |  |  |  |  | 0 |
| Unknown severity | 0 | 0 |  |  |  |  | 0 |

^1^ Health neglect was not counted, but rated as present/absent over the course of four months.

Table S8.

*Hierarchical Logistic Regression of Lifetime History and Final Risk Judgment in Prediction of Adverse Outcomes During a Four-Months Follow-Up*

|  | B(SE) | *p* | Odds Ratio | 95% CI | Chi-square | df | *p* | Nagelkerke R^2^ |
| --- | --- | --- | --- | --- | --- | --- | --- | --- |
| **Substance Use (*N* = 103)** | |  |  |  |  |  |  |  |
| Block 1 |  |  |  |  | 21.51 | 1 | .000 | .253 |
| History | 2.20 (.52) | .000 | 9.06 | [3.24, 25.32] |  |  |  |  |
| Block 2 |  |  |  |  | 24.38 | 3 | .000 | .283 |
| History | 1.52 (.65) | .020 | 4.58 | [1.28, 16.39] |  |  |  |  |
| Final Risk Judgment (Moderate v. low) | 0.99 (.67) | .141 | 2.68 | [0.72, 9.98] |  |  |  |  |
| Final Risk Judgment (High v. low) | 1.00 (.63) | .111 | 2.72 | [0.80, 9.28] |  |  |  |  |
| ΔR^2^ |  |  |  |  | 2.87 | 2 | .238 |  |
|  |  |  |  |  |  |  |  |  |
| **Self-Injury (*N* = 104)** |  |  |  |  |  |  |  |  |
| Block 1 |  |  |  |  | 15.59 | 1 | .000 | .187 |
| History | 1.65 (.44) | .000 | 5.22 | [2.23, 12.24] |  |  |  |  |
| Block 2 |  |  |  |  | 18.94 | 3 | .000 | .224 |
| History | 1.13 (.66) | .089 | 3.09 | [0.84, 11.33] |  |  |  |  |
| Final Risk Judgment (Moderate v. low) | .25 (.75) | .738 | 1.28 | [0.30, 5.54] |  |  |  |  |
| Final Risk Judgment (High v. low) | 1.58 (.98) | .108 | 4.84 | [0.71, 33.08] |  |  |  |  |
| ΔR^2^ |  |  |  |  | 3.35 | 2 | .187 |  |
|  |  |  |  |  |  |  |  |  |
| **Victimization (*N* = 102)** |  |  |  |  |  |  |  |  |
| Block 1 |  |  |  |  | 4.28 | 1 | .039 | .056 |
| History | 1.25 (.61) | .042 | 3.49 | [1.05, 11.62] |  |  |  |  |
| Block 2 |  |  |  |  | 7.96 | 3 | .047 | .103 |
| History | 0.57 (.71) | .422 | 1.77 | [0.44, 7.09] |  |  |  |  |
| Final Risk Judgment (Moderate v. low) | 1.03 (.61) | .089 | 2.81 | [0.85, 9.27] |  |  |  |  |
| Final Risk Judgment (High v. low) | 0.96 (.56) | .087 | 2.62 | [0.87, 7.89] |  |  |  |  |
| ΔR^2^ |  |  |  |  | 3.68 | 2 | .159 |  |
